# Serum albumin oxidation and inter-alpha-trypsin-inhibitor heavy chain-4 abundance correlate with the dystropathology in the rat model of Duchenne muscular dystrophy

**DOI:** 10.1042/BCJ20260160

**Published:** 2026-10-27

**Authors:** Angelo Patrick R. Bautista, Aude Lafoux, Irene Tsioutsias, Jessica R. Terrill, Caroline Le Guiner, Corinne Huchet, Peter G. Arthur

**Affiliations:** 1 School of Molecular Sciences, University of Western Australia, Perth, WA, Australia; 2 Therassay Platform, Nantes Université, Nantes, France; 3 School of Human Sciences, University of Western Australia, Perth, WA, Australia; 4 TaRGeT Lab, Nantes Université, Nantes, France

**Keywords:** albumin oxidation, biomarkers, Duchenne muscular dystrophy, inflammation, inter-alpha trypsin inhibitor 4, oxidative stress

## Abstract

Duchenne muscular dystrophy (DMD) is a fatal X-linked genetic disease caused by the absence of functional dystrophin, resulting in muscle membrane fragility, chronic inflammation, and progressive muscle wasting. The lack of effective treatments is further compounded by the slow onset of measurable changes in clinical outcomes, such as muscle strength, which often take a year or longer to become evident. Blood-based biomarkers that can predict treatment efficacy and monitor disease progression would significantly accelerate therapeutic development. We propose that oxidative modifications to proteins in blood, a consequence of muscle inflammation, could be used to monitor disease progression in DMD. Using the *DMD^mdx^* rat, a model with more severe dystropathology than the mouse model of dystrophy (*mdx*), we assessed inflammation and oxidative stress in muscle and blood across disease stages. *DMD^mdx^* rats aged 1.5–12 months showed sustained immune activation, as indicated by elevated circulating leukocytes and increased myeloperoxidase content in dystrophic muscle compared with wild type. Oxidative protein modifications, including carbonylation, tyrosine chlorination, and thiol oxidation, were significantly elevated in serum and tissues of dystrophic rats. Two proteins showed distinct disease-associated oxidative changes: albumin, which exhibited consistently increased thiol oxidation across ages, and inter-alpha-trypsin inhibitor 4 (ITIH4), which showed elevated chlorination between 1.5 and 4.5 months. Notably, serum ITIH4 abundance correlated with markers of muscle inflammation and oxidative stress. These findings identify serum albumin thiol oxidation and serum ITIH4 abundance as promising blood-based biomarkers for monitoring inflammation-associated dystropathology in DMD.

## Introduction

Duchenne muscular dystrophy (DMD) is a fatal X-linked genetic disease that affects 1 in 5000 male births worldwide [1]. Genetic mutations in the dystrophin gene result in progressive muscle wasting, which is caused by an absence of functional dystrophin, a structural muscle protein [2]. Loss of dystrophin compromises the stability of the sarcolemma, which results in muscle that is susceptible to damage during contraction [3]. As a consequence of ongoing damage, there is a loss of muscle mass and replacement of muscle tissue with fat and fibrotic tissues [4]. Muscle damage in DMD is associated with chronic inflammation, which is considered to be a major contributor to muscle loss [5]. The role of inflammation in the progression of dystropathology has led to investigations into the potential of anti-inflammatory drugs to delay muscle degeneration and disease progression [6,7].

One of the many challenges of drug development in DMD, however, is that clinical outcome measures utilized in trials are based on muscle strength and therefore require long treatment times (at least 6 months) to have a measurable result [8,9]. These clinical outcome measures include the North Star Ambulatory Assessment, the 6-min walk test, motor function measure domain 1, and performance of upper limb [9]. There is therefore a need to identify biomarkers of dystropathology that could act as early indicators of treatment effectiveness. More specifically, blood-based biomarkers would help speed up the assessment of treatment effectiveness, as this biofluid can easily be collected to monitor early signs of drug benefit.

We have previously hypothesized that measures of inflammation and associated oxidative stress have the potential to serve as monitoring biomarkers of dystropathology [10]. In particular, we have focused on readouts of reactive oxygen species generation by immune cells. In inflammatory conditions such as DMD, chronic inflammation is associated with oxidative stress, as immune cells such as neutrophils can generate a wide array of oxidants such as superoxide, hydrogen peroxide, and hydroxyl radicals [11,12]. In addition to this, neutrophils also contain the enzyme myeloperoxidase (MPO), which can catalyze the formation of hypochlorous acid (HOCl) [13–15]. The formation of HOCl can also result in the formation of reactive chloramines [15]. These oxidants can cause oxidative modifications to proteins, such as tyrosine chlorination, protein thiol oxidation, and carbonylation, which can then be used as readouts of oxidant generation in tissue [16].

We have previously investigated protein oxidation in animal models of DMD and have shown increased albumin thiol oxidation in the mouse model of dystrophy *(mdx)* [17,18] and increased tyrosine chlorination in both the *mdx* mice and the *GRMD* dog model of dystrophy [19,20]. Recently, a rat model for DMD was established, *DMD^mdx^* [21]. Dystrophic rats have undetectable tissue dystrophin, exhibit myofiber necrosis, progressive loss of muscle mass, increased inflammatory cell infiltration, and progressive dilated cardiomyopathy [21,22]. The *DMD^mdx^* rat is of interest for preclinical studies because it exhibits a severe dystropathology that better resembles the severity of human dystropathology, compared with the milder phenotype of the *mdx* mouse model [21,22].

The *DMD^mdx^* rat is a promising alternative to the *mdx* mouse, but there has been limited previous research into oxidative stress in this model. Previous studies have shown increased mRNA expression of oxidative stress markers in lung endothelial cells as well as an increase in total protein thiol oxidation in muscle [23,24]. In the present study, we first aimed to provide evidence of inflammation and oxidative stress in *DMD^mdx^* muscle and serum. To test this, we tracked the progression of dystropathology, inflammation and oxidative stress in *DMD^mdx^* rats from ages 1.5–12 months. This involved measuring oxidative modifications to proteins, including thiol oxidation, carbonylation, and protein tyrosine chlorination. Thiol oxidation and carbonylation are modifications that can be caused by a wide array of oxidants [25]. In contrast, tyrosine chlorination is a specific marker of neutrophil activation as its formation is dependent on the neutrophil enzyme, MPO [13,14].

Since we have previously hypothesized that neutrophil-mediated oxidative stress has a significant role in dystropathology [17,18,26,27], a secondary aim of the present study was to identify serum proteins that have increased tyrosine chlorination in *DMD^mdx^* rats. In the present study, we established the presence of inflammation and oxidative stress in *DMD^mdx^* muscle and have identified two serum proteins undergoing oxidative modifications that correlate with pathology, inflammation and oxidative stress in dystrophic muscle. The present study provided evidence of oxidative stress in *DMD^mdx^* and identified inter-alpha-trypsin inhibitor 4 (ITIH4) as a novel serum inflammatory biomarker for DMD.

## Results

### Dystropathology of the rat model of dystrophy (DMD^mdx^)

#### Blood markers of inflammation

We observed an ongoing inflammatory response in *DMD^mdx^* blood compared with wild type. Leukocyte concentration was up to two-fold higher in *DMD^mdx^* rats from 3–7 months, peaking at 3 months (Figure 1A). This increase in leukocytes was primarily due to an increase in the neutrophil population from 1.5–7 months of age (Figure 1B). The neutrophil population was also highest at 3 months, reaching up to 44 ± 2% of total leukocytes (Figure 1D). Platelet count was also up to 1.6-fold higher at all ages of *DMD^mdx^* (Figure 1C).

**Figure 1:**
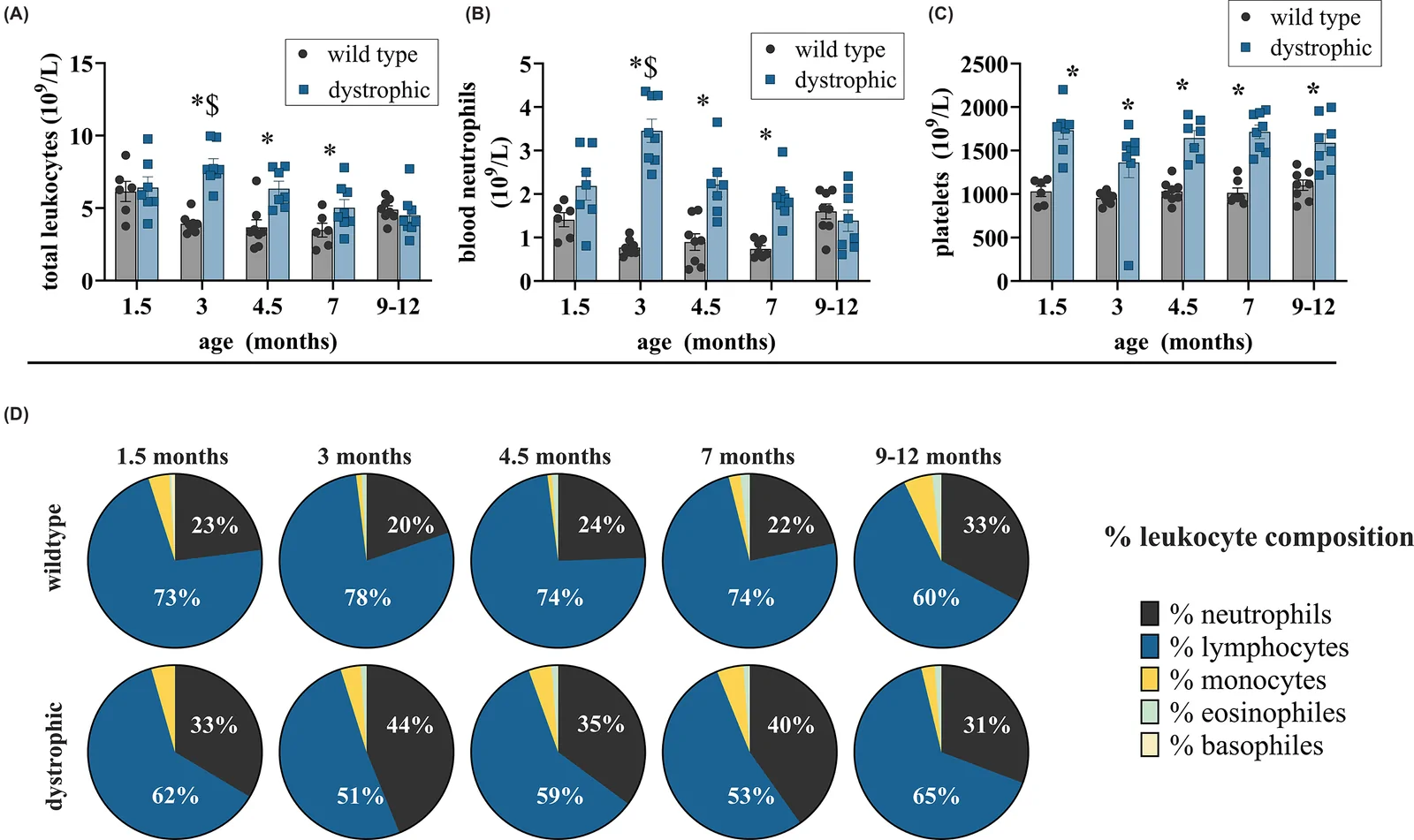
Blood leukocytes and platelet count in the DMDmdx Blood leukocytes and platelet count in the *DMD^mdx^*. (**A**) Blood leukocytes, (**B**) neutrophil content, (**C**) platelets, and (**D**) leukocyte composition of wild-type and dystrophic rats. * = significant difference between wild-type and dystrophic rats in the same age group, $ = significant difference between age groups of dystrophic rats, *P<*0.05. *n =* 6–8, error bars represent the mean ± SEM.

#### Tissue markers of inflammation and muscle physiology

Tissue neutrophil abundance, as measured by MPO content, increased in *DMD^mdx^* muscle at all ages compared with wild type, reaching a level 8.5-fold higher at its peak at 3 months (Figure 2A). MPO content dropped after 3 months in *DMD^mdx^* rats only. Physiological assessment of grip strength showed 36.1 ± 4.2% lower force output in *DMD^mdx^* rats compared with wild type (Figure 2B). While there was no difference in specific grip strength between different ages of *DMD^mdx^* rats, there was an observed decrease in force output in wild type after 1.5 months (Figure 2B).

**Figure 2:**
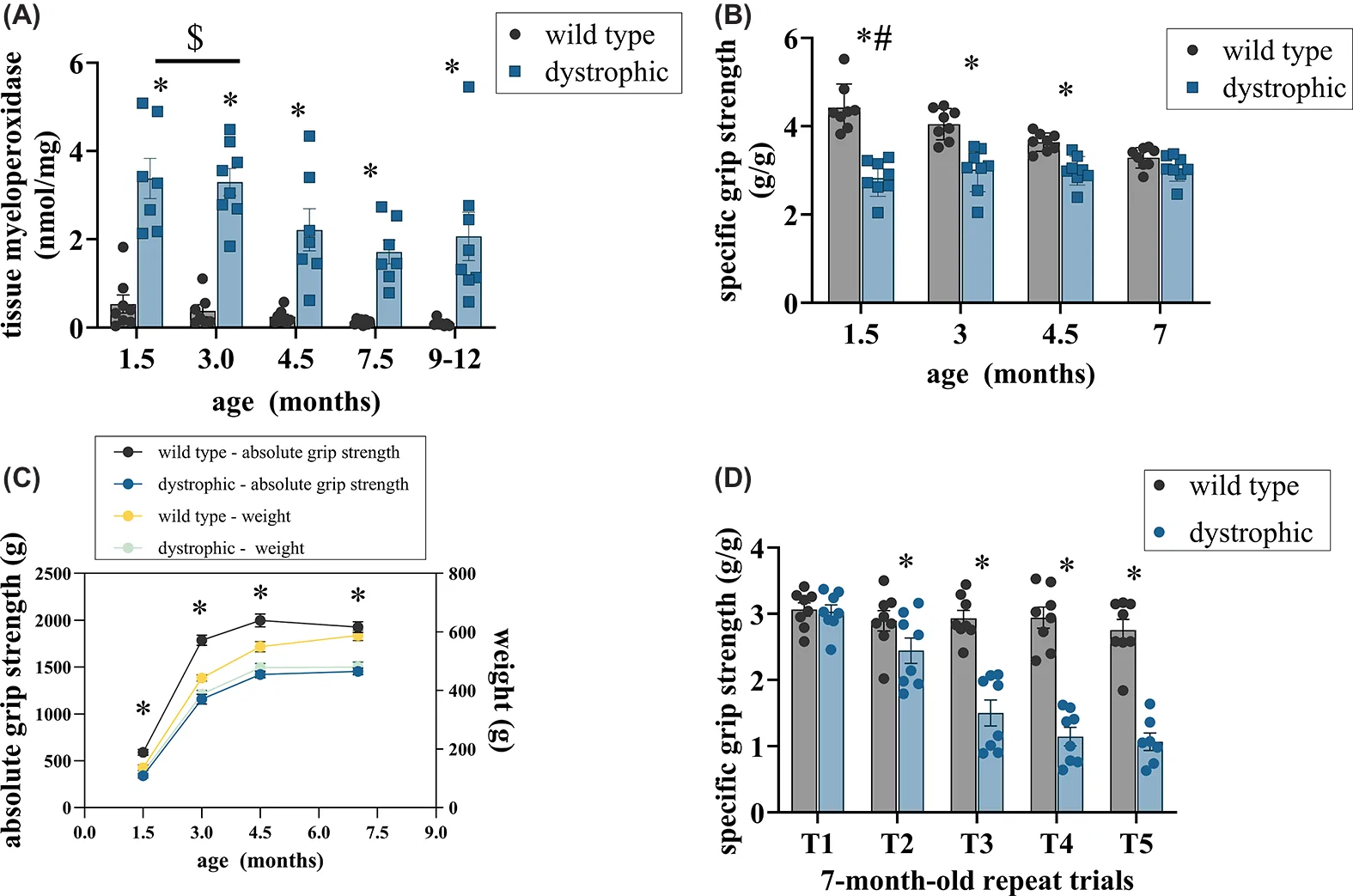
Muscle tissue markers of inflammation and muscle strength in the DMDmdx Muscle tissue markers of inflammation and muscle strength in the *DMD^mdx^*. (**A**) Tissue MPO, *n =* 7–8; (**B**) specific grip strength, *n =* 8; (**C**) absolute grip strength and body weight, *n =* 7–8; (**D**) change in specific grip strength in five consecutive trials. * = significant difference between wild-type and dystrophic rats of the same age, $ = significant difference between age groups in dystrophic rats, # = significant difference between age groups in wild-type rats, *P<*0.05. Error bars represent mean ± SEM.

The apparent decrease in specific force in wild-type animals does not indicate developing muscle weakness. Instead, it reflects the animals’ increase in body weight over time (Figure 2C). Specific grip strength is calculated by normalizing absolute grip strength to body weight. As body weight increases, the normalized force value can appear lower even when there is no decrease in absolute force production. Repeated grip strength trials in 7-month-old animals did not show a decline in force output in wild-type animals, unlike the pattern observed in dystrophic animals (Figure 2D). This suggests that the wild-type animals did not develop measurable muscle fatigue across the trials compared with dystrophic animals.

#### Serum markers of muscle membrane leakage

Serum biochemical analysis indicated muscle membrane leakage and a potential disturbance of creatine metabolism in *DMD^mdx^*. Serum creatine kinase (CK), a widely used marker of muscle membrane leakage, was up to 29-fold higher in *DMD^mdx^* rats compared with wild type from 1.5–4.5 months, returning to control levels by 7 months (Figure 3A). High serum creatine and low serum creatinine concentrations have been previously shown to be potential serum metabolic biomarkers of DMD [28,29]. Serum creatine concentration was up to three-fold higher in *DMD^mdx^* rats compared with wild type from 1.5–4.5 months, returning to control levels by 7 months (Figure 3B). Serum creatinine was up to 1.4-fold lower in *DMD^mdx^* rats compared with wild type from 3–12 months (Figure 3C). While there was no difference in serum creatine between different ages of *DMD^mdx^* rats, serum creatinine was higher at ages 3–7 months in wild-type rats compared with other ages.

**Figure 3:**
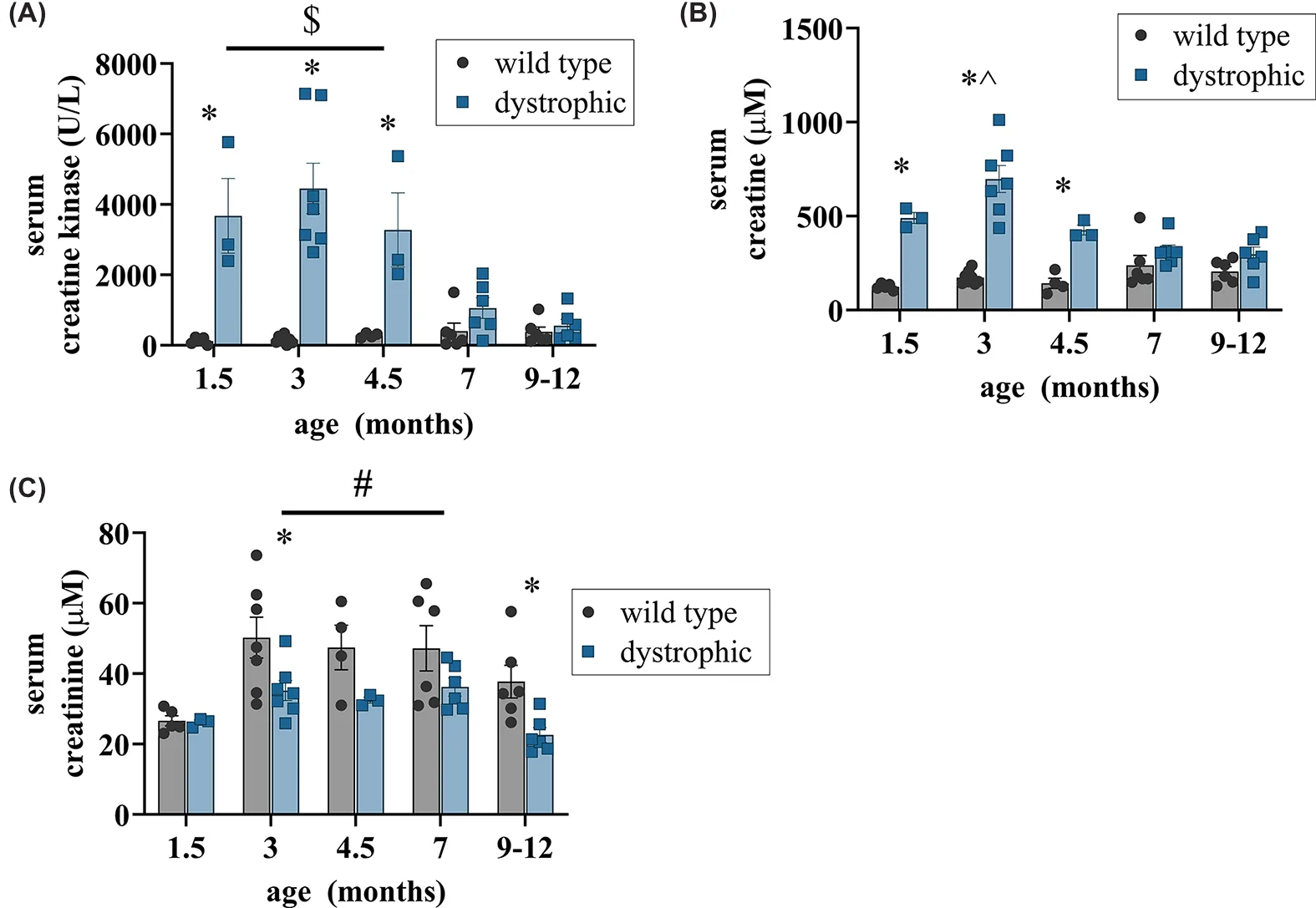
Serum markers of muscle membrane leakage in DMDmdx Serum markers of muscle membrane leakage in *DMD^mdx^*. (**A**) Serum CK, *n =* 3–8; (**B**) serum creatine, *n =* 3–8; (**C**) serum creatinine, *n =* 3–7. * = significant difference between wild-type and dystrophic rats of the same age; $= significant difference between age groups of dystrophic rats; #= significant difference between age groups of wild-type rats; 3–7 months old are significantly higher from other age group of the wild-type strain, *P<*0.05. Data represent mean ± SEM.

### Oxidative modification to tissue proteins in DMD^mdx^

Given the increased presence of neutrophils in dystrophic tissues, we investigated whether this was associated with increased oxidative stress. Protein thiol oxidation, carbonylation, and tyrosine chlorination are oxidant-induced post-translational modifications that can occur as a consequence of oxidative stress. Tissue thiol oxidation was up to 39.9 ± 12.0% higher in *DMD^mdx^* at all ages (Figure 4A) with carbonylation and tyrosine chlorination higher at ages 1.5–3.0 months (Figure 4B,C).

**Figure 4:**
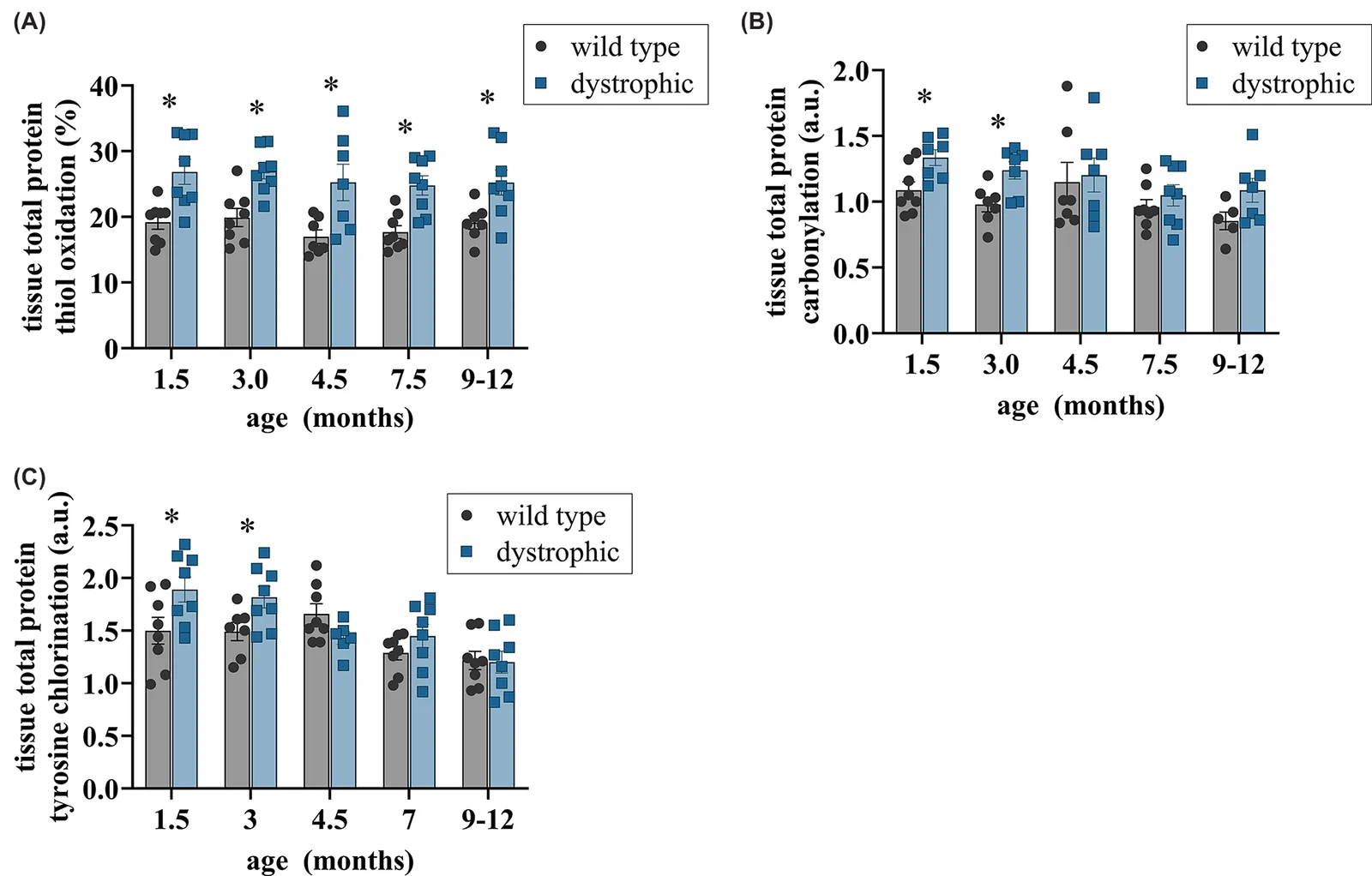
Oxidative modifications to muscle tissue (pectoralis) proteins in DMDmdx Oxidative modifications to muscle tissue (pectoralis) proteins in *DMD^mdx^*. (**A**) Protein thiol oxidation, *n =* 7–8; (**B**) carbonylation, *n =* 5–8; (**C**) tyrosine chlorination, *n =* 7–8. * = significant difference between wild-type and dystrophic rats of the same age, *P<*0.05. Error bars represent ± SEM.

### Oxidative modifications to serum proteins in DMD^mdx^

To determine whether oxidative stress in tissues was reflected in serum, we measured serum albumin thiol oxidation and protein carbonylation. Due to limited samples, only three ages of rats (1.5, 3, and 4 months) were able to be analyzed. Albumin thiol oxidation is a sensitive marker of oxidative stress that has been found to be elevated in a plethora of inflammatory diseases [30–34]. Serum albumin thiol oxidation was up to 18.64 ± 8.21% higher in *DMD^mdx^* from ages 1.5 to 3 months (Figure 5A). Serum protein carbonylation was found to be lower in dystrophic rats at age 4.5 months, with no differences observed in other age groups (Figure 5B).

**Figure 5:**
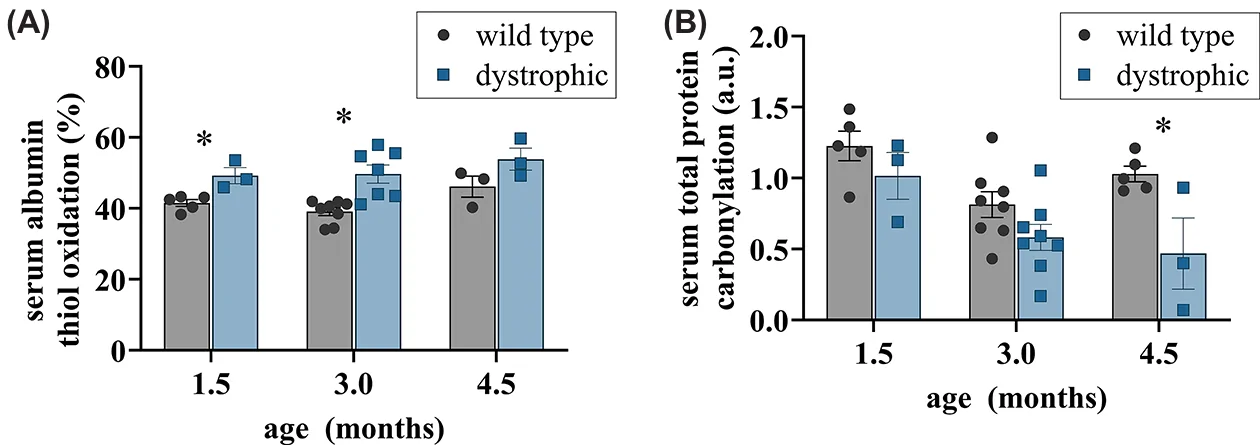
Oxidative modifications to serum proteins in DMDmdx Oxidative modifications to serum proteins in *DMD^mdx^*. (**A**) Serum albumin thiol oxidation, *n =* 3–8. (**B**) Serum carbonylation, *n =* 3–8. * = significant difference between wild-type and dystrophic rats of the same age, *P<*0.05. Error bars represent ± SEM.

### Identification of serum proteins with chlorinated tyrosines

As there was evidence of chlorinated protein tyrosines in tissues, we next examined whether this modification was also present in the serum of dystrophic rats and performed mass spectrometry (MS) to identify specific proteins. Immunoblotting for chlorinated tyrosines (ClTyr) detected three proteins, which were then identified by MS (Figure 6A–C). Serum proteins identified were (1) ITIH4, 120 kDa; (2) albumin, 70 kDa; and (3) gc-globulin, 50 kDa; Supplementary Mass Spectrometry data (Supp. MS).

**Figure 6:**
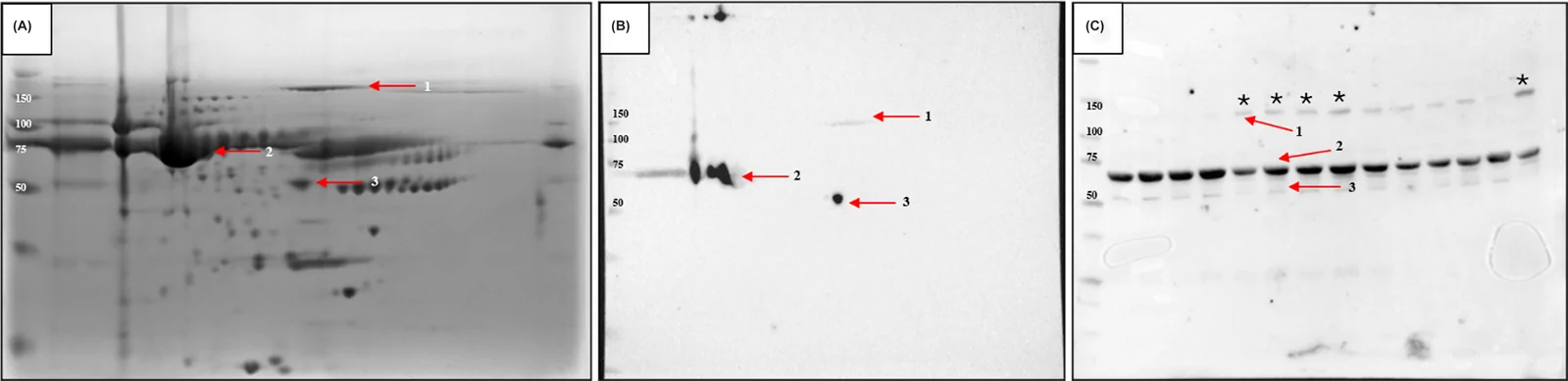
Immunoblot for protein ClTyr in the serum of 3-month-old DMDmdx Immunoblot for protein ClTyr in the serum of 3-month-old dystrophic rats. (**A**) 2D gel, (**B**) 2D western blot, and (**C**) 1D western blot. Proteins identified by MS are (1) ITIH4, (2) albumin, and (3) gc-globulin. (*) indicates dystrophic rat serum.

MS identified peptides containing ClTyr in albumin and ITIH4 but not in gc-globulin (Table 1). Albumin and ITIH4 were further investigated as we confirmed the presence of chlorinated peptides in these proteins (Supplementary Figures S1 and S2). Further validation was performed using a dye-affinity pulldown with Cibacron Blue, which enriched albumin and ITIH4 and yielded a purified fraction containing chlorinated species at molecular weights consistent with these proteins (Supplementary Figures S3 and S4).

**Table 1:** Serum proteins with chlorinated tyrosines identified by mass spectrometry

Protein	Observed molecular weight (kDa)	Number of peptide matches	Unique peptide sequences	Significant peptide with ClTyr
ITIH4	120	146	46	RLGM**Y_Cl_**ELLLK
Albumin	70	153	20	LGE**Y_Cl_**GFQNAILVR
Gc-globulin	50	18	7	-

### Albumin tyrosine chlorination and total albumin is elevated in tissues of dystrophic rats

To determine the extent of albumin modification in dystrophy, albumin concentration and chlorination were quantified in serum and tissue. Since neutrophil activity was highest at three months (Figures 1 and 2), this time point was used for analysis. While there was no difference in albumin chlorination in the serum of *DMD^mdx^* rats compared with wild type (Figure 7A), tissue albumin chlorination was found to be 4.8-fold higher in *DMD^mdx^* rats compared with wild type (Figure 7B). We also quantified albumin content in the serum and tissue of *DMD^mdx^* rats; albumin content was 15.32 ± 0.02% lower in *DMD^mdx^* serum and 44.15 ± 0.05% higher in *DMD^mdx^* muscle compared with wild type (Figure 7 C,D). Total protein gels and western blot membranes are shown in Supplementary Figures S5–S8.

**Figure 7:**
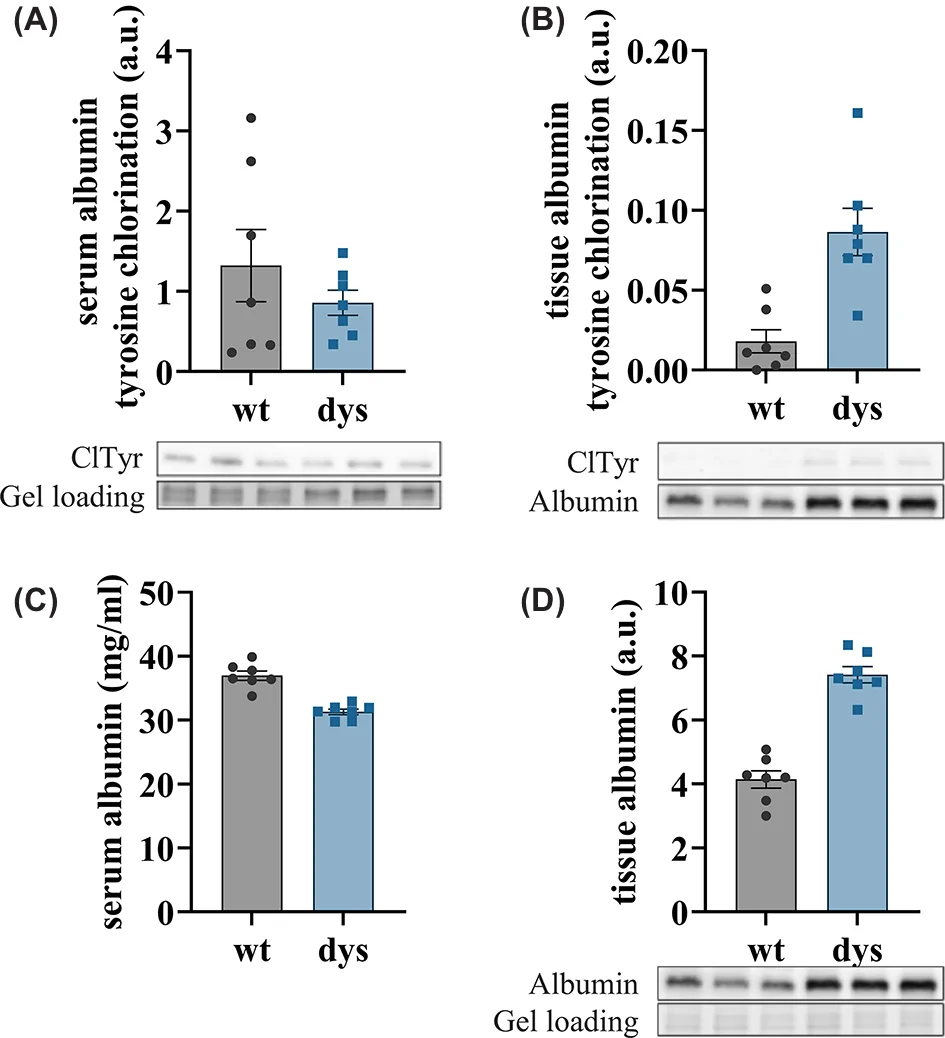
Albumin concentration and tyrosine chlorination in serum and tissue of 3-month-old rats Albumin concentration and tyrosine chlorination in serum and tissue of 3-month-old rats. (**A**) Serum albumin tyrosine chlorination, (**B**) tissue albumin tyrosine chlorination, (**C**) serum albumin concentration, and (**D**) tissue albumin content. *= significant difference, *P<*0.05. Error bars represent ± SEM, *n =* 7. Presented blots are representatives of the replicates.

### Inter-alpha-trypsin inhibitor 4 (120 kDa) is more abundant in serum and tissues of dystrophic rats

ITIH4 exists as a combination of an intact protein (120 kDa) and fragments ranging from 37–100 kDa [35–37]. The following analyses focused on the 120 kDa protein, as there was no ClTyr signal that corresponded to the fragments. Since the ClTyr western blot showed that a 120 kDa protein signal is up to four-fold higher in *DMD^mdx^* rats (Figure 8A), we first investigated whether the increase in this signal from ClTyr was a consequence of a relative increase in chlorination of ITIH4 or due to changes in the concentration of total ITIH4. The concentration of ITIH4 was up to two-fold higher in the serum of *DMD^mdx^* rats aged 1.5–7 months, compared with wild type (Figure 8B). There was also an observed increase in ITIH4 abundance in wild-type rats after 9 months (Figure 8B). Ratio analysis of chlorinated ITIH4 to total ITIH4 showed significant chlorination in 1.5- and 4.5-month-old *DMD^mdx^* rats compared with wild type (Figure 8C). These data suggest that changes in the signal of ClTyr were generally a consequence of changes in ITIH4 abundance. Muscle from 3-month-old rats was analyzed to investigate ITIH4 abundance and its chlorination. Intact ITIH4 was up to 18-fold higher in *DMD^mdx^* muscle, but no chlorination was observed (Figure 8D). Western blot results for all time points are shown in Supplementary Figure S9.

**Figure 8:**
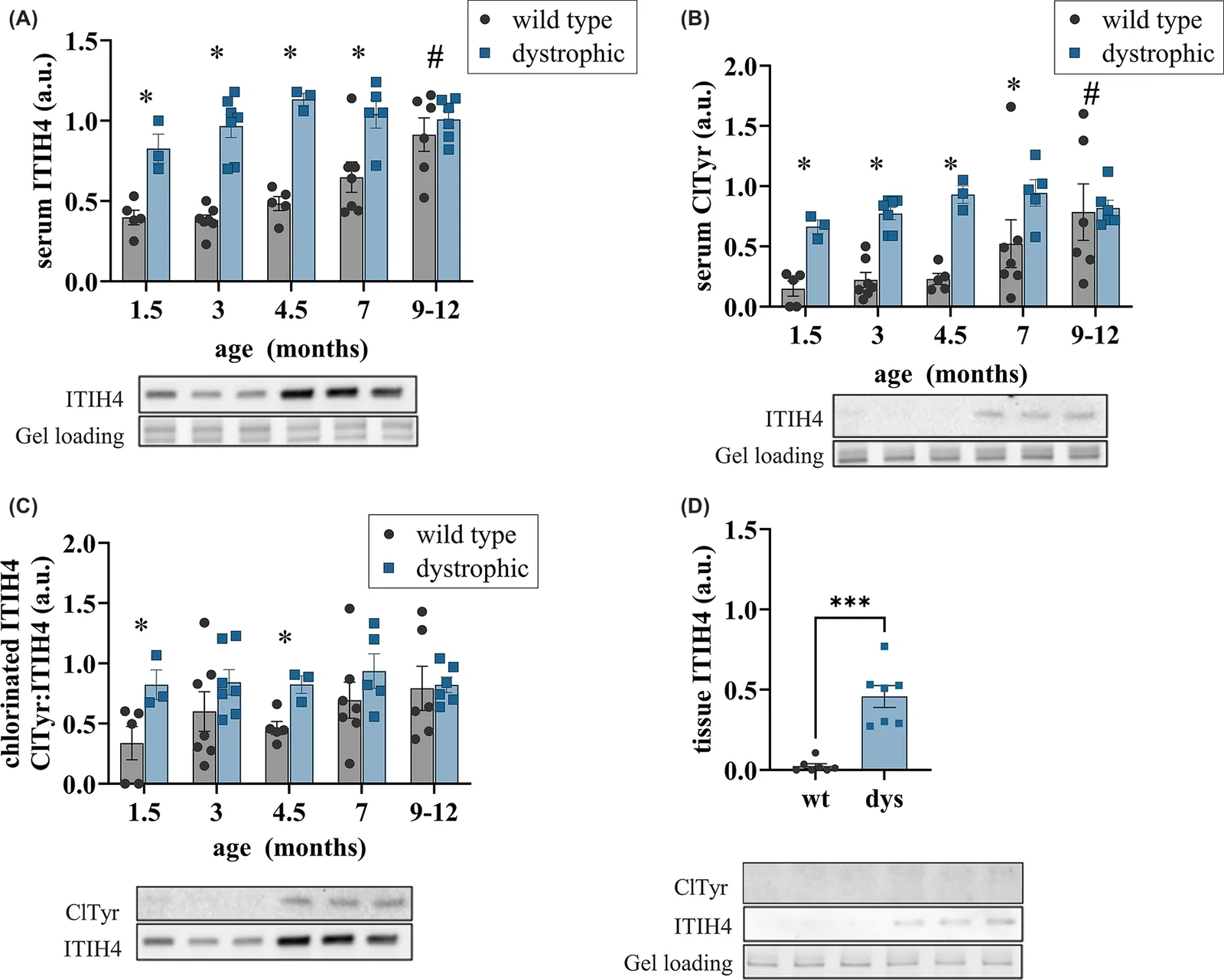
ITIH4 abundance and chlorination in dystrophic (dys) and wild-type (wt) rats ITIH4 abundance and chlorination in dystrophic (dys) and wild-type (wt) rats. (**A**) Serum ClTyr, *n =* 3–7; (**B**) serum ITIH4 abundance, *n =* 3–7; (**C**) ratio analysis of serum ClTyr and ITIH4, *n =* 3–7; (**D**) tissue ITIH4 abundance, *n =* 7. *= significant difference between control and dystrophic rats; # = significant difference between age groups of wild-type rats, *P<*0.05. Error bars represent mean ± SEM. Blots presented are from 3–4.5 months old rats. Presented blots are representatives of the replicates.

### Correlation of serum markers (albumin thiol oxidation and serum ITIH4) with dystropathology markers

Correlation analysis was performed between serum ITIH4 and albumin thiol oxidation and dystropathology markers to determine whether there was a direct relationship between these markers. There was a significant positive correlation between the serum markers (ITIH4 and albumin thiol) and neutrophil content in blood and tissues, serum CK, serum creatinine, and tissue thiol oxidation (Table 2). A significant negative correlation was found between ITIH4 and serum creatinine. Additionally, a positive correlation between albumin thiol oxidation and ITIH4 abundance indicated a potential relationship between the identified serum markers. The correlation between ITIH4, albumin thiol oxidation, and dystropathology markers indicated that the identified serum markers reflect dystropathology in *DMD^mdx^* rats.

**Table 2:** Correlation of serum markers (albumin thiol oxidation and ITIH4) with dystropathology markers

Identified serum markers	Dystropathology markers	*r*	*P*	*n*
Albumin thiol oxidation	Blood neutrophils	0.434*	0.024	27
	Tissue MPO	0.560*	0.002	27
	Tissue thiol oxidation	0.523*	0.003	29
	Serum CK	0.689*	<0.001	28
	Serum creatinine	−0.300	0.129	27
	Serum creatine	0.582*	0.001	28
ITIH4 abundance	Blood neutrophils	0.477*	<0.001	51
	Tissue MPO	0.563*	<0.001	51
	Tissue thiol oxidation	0.389*	0.004	53
	Serum CK	0.388*	0.004	51
	Serum creatinine	−0.431*	0.001	50
	Serum creatine	0.489*	<0.001	51
Albumin thiol oxidation	Serum ITIH4 abundance	0.765*	<0.0001	27

*= significant correlation, *n =* number of pairs, *r* = Pearson coefficient, *P* = *P*-value, significance at *P<*0.05.

## Discussion

DMD is characterized by progressive muscle degeneration resulting from the absence of functional dystrophin, leading to sarcolemmal instability, chronic inflammation, oxidative stress, fibrosis, and loss of muscle function [1–5]. The findings presented in the present study demonstrate that the rat model of dystrophy exhibits these pathological conditions and provide evidence of oxidative stress in this animal model. The presence of increased protein carbonylation, tyrosine chlorination, and protein thiol oxidation in skeletal muscle, together with elevated albumin thiol oxidation and ITIH4 chlorination in serum, indicates that oxidative stress is present in the *DMD^mdx^* rat model. These oxidative modifications occurred alongside increased neutrophil activity, reduced force production, elevated serum CK and creatine, and reduced serum creatinine, suggesting that oxidative damage is closely associated with inflammation, muscle degeneration, and loss of function.

A major strength of the *DMD^mdx^* rat model is its ability to reflect the progressive nature of DMD. Our temporal analyses demonstrated disease progression, characterized by progressive changes in circulating biomarkers. Similar to observations in patients with DMD, serum CK decreased with disease progression, likely reflecting the gradual loss of viable muscle fibers capable of expressing the protein [38,39]. Similarly, the decrease in serum creatine observed may indicate a decrease in muscle mass, as creatine is also stored in myofibers [40,41]. These results are consistent with previous reports on the progression of muscle degeneration in both humans and animals [42–44]. Further supporting muscle loss in *DMD^mdx^* is the low serum creatinine across ages. Low serum creatinine is associated with low muscle mass, as creatinine formation from creatine occurs mostly in the muscle [45,46]. Decreasing serum creatinine concentration with disease progression has been shown to occur in DMD and has been proposed to be a marker of disease severity [47,48]. Taken together, the changes in these biochemical markers, along with the previously reported muscle degeneration in this animal model [21], reflect the progressive pathology characteristic of DMD.

Inflammation is known to result in oxidative stress due to the ability of immune cells, such as neutrophils, to produce oxidants [49–52]. We hypothesized that oxidatively modified proteins increase in abundance as a consequence of neutrophil activation. The present study showed that increased neutrophil tissue infiltration was associated with higher levels of protein carbonylation, thiol oxidation, and tyrosine chlorination, supporting previous observations in *mdx* mice and GRMD dogs [19,26,27,30,53,54]. Distinct temporal patterns were observed among these oxidative modifications. Protein carbonylation and tyrosine chlorination were elevated primarily during the early stages of disease, whereas protein thiol oxidation remained elevated across all ages examined. One explanation for this difference is the greater reactivity of cysteine thiol groups toward HOCl compared with tyrosine or amino acid side chains that form carbonyl derivatives. MPO, an enzyme found in neutrophils that is responsible for the generation of HOCl [13,55], was highly abundant in muscles at ages 1.5 and 3 months, after which levels declined. This decrease in MPO concentration likely resulted in a decrease in HOCl generation and, therefore, carbonylation and tyrosine chlorination. The reason for the sustained protein thiol oxidation at all ages may relate to the reactivity of HOCl with thiols relative to tyrosine and amino acid side chains (lysine, arginine, threonine, and proline) that are known to form carbonyls [56,57]. The reported second-order rate constants of some of these amino acids with HOCl at physiological pH are as follows: cysteine (3.6 × 10^8^ M^−1^ s^−1^) >> lysine (5 × 10^3^ M^−^^1^ s^−^^1^) >> tyrosine (44 M^−1^ s^−^^1^) > arginine (26 M^−1^ s^−1^) [58]. The high reactivity of cysteine thiols with HOCl makes them particularly sensitive to changes in oxidative stress caused by neutrophil activity. Our results provide evidence that protein thiol oxidation is a particularly sensitive marker of protein oxidative stress compared with carbonylation and tyrosine chlorination.

ClTyr, an irreversible oxidative protein modification, have been reported to increase in abundance in various inflammatory conditions [26,59–63]. Consistent with these studies, we found elevated concentrations of ClTyr in muscles of *DMD^mdx^* rats. As there was evidence of increased protein tyrosine chlorination in *DMD^mdx^* muscle, we investigated whether proteins with increased chlorination were also present in serum. Three serum proteins with ClTyr were detected by immunoblotting and identified by MS to be albumin, ITIH4, and gc-globulin. Among these, the chlorinated peptides were identified in albumin and ITIH4 but not in gc-globulin. The ratio of chlorinated protein to total protein (for albumin and ITIH4, as we confirmed the presence of chlorinated peptides) was quantified to determine the extent of protein chlorination. There was no significant difference in albumin chlorination in serum, although we did find increased albumin chlorination in *DMD^mdx^* muscle. The difference between serum and muscle may reflect degradation of chlorinated albumin in muscle, as irreversibly oxidized proteins have been reported to be more susceptible to proteolysis [64,65]. These data suggest that utilizing serum proteins with ClTyr may not be suitable biomarkers for investigating inflammation in dystrophy.

The detection of increased serum albumin thiol oxidation provides opportunities for biomarker development. Albumin was of particular interest because it contains a highly reactive free cysteine residue (Cys34) capable of undergoing oxidation [32]. Elevated serum albumin thiol oxidation suggests that albumin may act as a marker of oxidative stress occurring within dystrophic muscle [17,18,66]. Given the ability of albumin to exchange between tissues and blood [67,68], oxidation of albumin thiols may reflect exposure to an inflammatory and oxidizing microenvironment as the protein traffics through damaged muscle. Importantly, albumin thiol oxidation has also been reported in the *mdx* mouse model and DMD patients [17,18,66], strengthening its potential as a translational biomarker. The location where albumin undergoes thiol oxidation in dystrophy has not been established; however, it potentially undergoes oxidation as it transits through inflamed muscle, where it has been shown previously that albumin, oxidized thiols, and immune cells are colocalized [17,69]. Unlike tissue-based measurements, serum albumin oxidation could potentially provide a minimally invasive method for monitoring oxidative stress and treatment responses in DMD.

Another important finding was the identification of serum ITIH4 as a potential biomarker of dystrophic pathology. Although we initially detected increased chlorinated ITIH4 in dystrophic serum, subsequent analyses showed that the main change was increased total protein abundance rather than oxidation state. Elevated ITIH4 abundance was observed throughout much of the disease course, suggesting a sustained response to chronic pathology. While the biological role of ITIH4 in DMD remains unclear, previous reports have linked altered ITIH4 abundance to inflammatory conditions, fibrosis, neurodegenerative disease, and cancer [69–79]. The observation that ITIH4 accumulation has also been reported in fibrotic disorders [76,79] is particularly noteworthy given the progressive fibrosis characteristic of DMD [80,81]. It is therefore plausible that elevated ITIH4 reflects inflammatory or fibrotic remodeling processes in dystrophic muscle. Further research is needed to determine the role of ITIH4 in dystrophy and its biological function, as we also found that its abundance increases with age in wild-type rats. Nevertheless, ITIH4 protein abundance in serum may serve as a biomarker of inflammation in DMD.

Recently reported blood-based biomarkers of DMD have identified candidates that reflect multiple aspects of DMD pathology, including myofiber necrosis, inflammation, oxidative stress, motor performance, and fibrosis [82–84]. These findings emphasize that no single biomarker can fully capture disease progression and that a multimodal biomarker panel can provide more comprehensive information on changes in disease pathology. The biomarkers identified in the present study may complement existing and emerging DMD biomarkers. The strong positive correlation between serum albumin thiol oxidation and ITIH4 abundance observed in the *DMD^mdx^* rat may suggest that a common pathway, most likely inflammation, drives their upregulation. Inclusion of these markers in a biomarker panel alongside imaging modalities (MRI), functional assessments (ambulatory function and muscle tests), biochemical markers (CK and cytokines), and clinical parameters (age and weight) could help determine whether these proteins have predictive value for the assessment of DMD disease progression and treatment efficacy.

The present study also highlights the value of *DMD^mdx^* rat in investigating disease progression in DMD. Our results demonstrate a temporal relationship between inflammation, oxidative stress, and declining muscle function in the rat model. Although the *mdx* mouse remains the most widely used preclinical model, its phenotype is relatively mild compared with DMD patients [85,86]. In contrast, we and other researchers have shown that the *DMD^mdx^* rat exhibits more pronounced inflammation, muscle wasting, fibrosis, weakness, and progressive muscle function decline, characteristics that more closely resemble the human disease [21,22,87]. These distinctions are particularly important for biomarker studies because the progressive nature of pathology in the *DMD^mdx^* rat will allow longitudinal assessment of biomarkers across distinct disease stages.

In conclusion, we have shown an increase in inflammation and oxidative stress in the *DMD^mdx^* rat model, which is therefore a useful model for exploring these pathways in dystrophy. Disease progression, as determined by muscle degeneration, was also evident in the serum markers, which showed a similar progression that observed in DMD boys. Correlating with this disease progression, we identified two potential serum-based biomarkers that could be used as markers for monitoring inflammation and oxidative stress. Further studies are required to determine whether these markers are responsive to therapeutic interventions, and whether these markers exhibit the same response in humans.

## Materials and methods

### General reagents

All reagents were acquired from Sigma–Aldrich unless otherwise stated. Solutions and buffers used in assays were made up in double deionized water. Solutions and buffers used for MS were made up in MS grade water.

### Force measurement

Grip strength data used for these analyses were generated previously and are described in [21,88]. In brief, grip test measurements were performed by a researcher who was blind to genotype and treatment using a grip meter (Bio-GT3, BIOSEB, Vitrolles, France) attached to a force transducer. Repeated grip strength measurements were performed on the same rats were performed every 1.5 months from 1.5 to 7 months. Rats were placed with their forepaws on a T-bar and gently pulled backwards until they released their grip. Five trials were performed sequentially, and the results are expressed as grams (g) and normalized to body weight (g/g).

### Animal sampling

Before blood sampling, rats were anesthetized with etomidate (hypnomidate 2 mg/ml, Maphar, Casablanca, Morocco) and ketamine (Imalgene, 100 mg/ml, Merial, Ingelheim am Rhein, Germany). After blood sampling from the renal vein, rats were sacrificed by intravenous injection of pentobarbital (300 mg/kg, Dolethal, Vetoquinol UK Ltd, Buckingham, U.K.). Blood was collected for hematology analysis (to quantify leukocytes), and the remainder of the blood was allowed to clot to collect serum. The clot was removed by centrifugation at 2000×***g*** for 10 min. The supernatant was then immediately transferred into a microcentrifuge tube. Collected serum was stored at −80°C until analysis. Both biceps and pectoralis were dissected and either frozen in pre-cooled isopentane for histological analysis, or snap-frozen in liquid nitrogen for protein analysis.

### 2D-SDS/PAGE and protein identification by mass spectrometry

Serum samples for two-dimensional sodium dodecyl sulfate polyacrylamide electrophoresis (2D-SDS/PAGE) were diluted in a solution containing 7 M urea, 2 M thiourea, 2% (w/v) CHAPS, 1% (v/v) ampholyte, pH 3–10 (Bio-lyte/Bio-Rad), and 1 mM dithiothreitol (DTT). First-dimension focusing was done on a 7 cm pH 4–7 immobilized pH gradient strip (ReadyStrip-IPG, Bio-Rad) using IPGphor Isoelectric Focusing System (Amersham Pharmacia Biotech). Second-dimension separation was performed on 10% polyacrylamide gels. Protein gels were either immunoblotted for ClTyr (1:500; Hycult Biotech-HP5002) or stained with Coomassie blue for protein spot excision.

Gel protein spots corresponding to proteins labeled with the ClTyr antibody were excised and destained with 25 mM ammonium bicarbonate in 50% acetonitrile (ACN) at 37°C for 45 min. Gel pieces were then dried by using a centrifugal vacuum concentrator. Protein was digested by the addition of 125 ng trypsin in 25 mM ammonium bicarbonate. The digestion reaction proceeded at 37°C for 16 h. Digested protein was extracted by the addition of 1% trifluoroacetic acid (TFA) in ACN and incubation at room temperature for 20 min. Extracts were pooled and desiccated with a centrifugal vacuum concentrator. Extracts were reconstituted in 20 μl of 2% ACN, 0.1% formic acid (FA) solution for loading into a Prominence (Shimadzu) HPLC system for chromatographic separation, which was directly sprayed into a 5600 TripleTOF^™^ (Sciex) mass spectrometer. The HPLC mobile phase consisted of 0.1% FA and 2% ACN in water (A) and 0.1% FA and 98% ACN in water (B). Gradient elution was performed with 2% B for 3 min, increased to 40% B at 15 min, then ramped to 98% B by 16 min and held for 1 min before reduced back to 2% B within 1 min. Column temperature was 40°C. Positive electrospray ionization mode was operated to acquire MS data by information dependent acquisition, where only the top 20 MS peaks between 400 and 1250 m/z were selected for further MS/MS scan. The output .wiff files containing MS and MS/MS spectra were submitted to the MASCOT database for identification of peptides, using the following search conditions: UP2494_R_norvegicus (AA) database, trypsin digest with allowance for up to one missed cleavage per peptide, no fixed modifications, variable modification of chlorination of tyrosine residues, MS tolerance of 0.2 Da and MS/MS tolerance of 0.2 Da. Proteins were identified on the basis of two or more peptides with ion scores exceeding the significance threshold.

### Serum and tissue sample preparation for SDS/PAGE and immunoblotting

Serum samples were diluted using sample buffer (0.5 M Tris, pH 7.0, with 0.5% w/v SDS). Diluted serum samples were further diluted with 2× Laemmli buffer (125 mM Tris, pH 6.8, 4% SDS, 20% w/v glycerol, 0.02% bromophenol blue, and 200 mM DTT) and heated for 10 min at 80°C before loading onto a 4%–15% gradient stain-free polyacrylamide gel (Bio-Rad). Tissues (pectorales) were homogenized in a sample buffer supplemented with protease inhibitor (Roche). Insoluble components were separated by centrifugation. The protein concentration of supernatants was determined by detergent-compatible protein assay (DC Assay, Bio-Rad), with the concentrations then adjusted to 2 mg/ml for each extract. Extracted tissue proteins were diluted with 2× Laemmli buffer and processed the same way as serum for SDS/PAGE. Gels were imaged using the ChemiDoc MP Imaging System.

Proteins were transferred to a nitrocellulose membrane using a Trans-Blot Turbo Transfer System (Bio-Rad). Blocking buffer was 5% skim milk inTris-buffered saline with Tween 20 (TBST—10 mM Tris, pH 7.0, 150 mM NaCl, 0.1% v/v tween 20). Chlorotyrosine antibody 1:500 (Hycult Biotech, HP5002) and ITIH4 antibody 1:1000 (Cloud-Clone Corp., PAH776Hu03) were diluted in TBST containing 5% bovine serum albumin (BSA). Albumin antiserum (Sigma–Aldrich, A0433) was diluted 1:10,000 in TBST with no BSA.

Membranes were incubated with primary antibody overnight at 4°C. Secondary antibody probing was performed using a horseradish peroxidase-conjugated goat anti-rabbit (#31460, Thermo Fisher Scientific) diluted (1:10000) in blocking buffer. Secondary antibody incubation was performed at room temperature for 1 h. Detection was performed by addition of enhanced chemiluminescence reagent (Bio-Rad), and the image was captured using a ChemiDoc MP Imaging System (Bio-Rad). Band analysis was performed using ImageJ software (NIH) [89]. Band signal intensity for each antibody was normalized to full lane protein signal. Further analysis of protein chlorination was performed by calculating the ratio of halogenated protein signal (chlorotyrosine) to total protein signal (ITIH4 or albumin). To minimize variability between membranes when analyzing chlorinated ITIH4, gels were cut between 100 and 150 kDa and transferred onto the same membrane. Validation of chlorotyrosine antibody and representative gels and membranes used for ratiometric calculations are shown in Supplementary Figures S10 and S11.

### Albumin thiol oxidation in serum

Serum albumin thiol oxidation was measured using capillary electrophoresis [66]. In brief, serum samples were incubated for 5 min with 30 mM maleimide–polyethylene glycol (5 kDa; JenKem Technology) in 40 mM imidazole, pH 7.4. Incubated serum samples were diluted with 45 mM phosphate buffer (pH 8.0), 0.01% v/v DMSO with 2.5% w/v SDS before loading onto an Agilent 7100 capillary electrophoresis system. The background electrolyte was composed of 45 mM phosphate buffer (pH 8.0) with 2.5% w/v SDS. The capillary was a 50-µm bare fused silica with an effective length of 32.5 cm. Samples were injected hydrodynamically (10 mbar for 10 s), protein migration was induced by applying a constant voltage of +15 kV. Peak detection was performed at 214 nm. The peaks were quantified as area under the curve using Agilent ChemStation software.

Serum albumin concentration was measured using capillary electrophoresis with the same parameters as described above. Rat serum albumin standard (Sigma–Aldrich, A6272) was used to construct the standard curve for serum albumin quantification.

### Tissue total protein thiol oxidation

Total tissue thiol oxidation was measured based on the 2-tag technique as previously described [90]. In brief, frozen tissue samples were crushed under liquid nitrogen, followed by homogenization in 20% trichloroacetic acid (TCA)/acetone. Precipitated proteins were separated from the homogenizing solution by centrifugation and washed with 100% acetone. Pellets were resuspended in sample buffer (0.5 M Tris, pH 7.0, with 0.5% w/v SDS) and incubated with the fluorescent dye BODIPY-FL-N-(2-aminoethyl)-maleimide (FLM; Invitrogen) to label the reduced thiols. Excess dye was quenched by the addition of cysteine before reduction of reversibly oxidized thiols by tris(2-carboxyethyl)phosphine. The resulting reduced thiols were labeled by a second fluorescent dye, Texas-Red-C2-maleimide (Invitrogen). Proteins were precipitated with 100% TCA, followed by 100% acetone to remove excess dye and resuspended in sample buffer. Samples were loaded into a 96-well plate for fluorometric analysis (Fluostar Optima). For FLM, wavelength excitation was set at 485 nm and emission at 520 nm. For Texas Red, the excitation wavelength was set at 595 nm and the emission wavelength at 610 nm. A standard curve for each dye was prepared using BSA. Results are presented as percent oxidized thiols.

### Protein carbonylation ELISA

Immunoassay-based detection of protein carbonyls was performed by derivatizing carbonyls with 2,4-dinitrophenylhydrazine to form 2,4-dinitrophenyl (DNP), which were then detected with an anti-DNP antibody [91]. Tissue samples were homogenized in PBS–EDTA, with extracted protein lysate diluted down to 10 μg/ml. Protein lysate (100 μl) was coated onto an ELISA plate overnight at 4°C. Plates were washed twice with PBS before the addition of 100 μl of 4 mM 2,4-dinitrophenylhydrazine (DNPH) in 4% TFA. Derivatization with DNPH was performed for 45 min at room temperature followed by washing with PBS–ethanol. Wells were blocked with 5% skim milk in PBS-1% Tween 20 (PBST) for 2 h at room temperature, followed by PBST wash. Anti-DNP antibody (1:10000—5% milk–PBST, D9656, Sigma–Aldrich) was added and incubated for 2 h at room temperature followed by PBST wash. Rabbit secondary antibody (1:10000—5% milk–TBST, #31460, Thermo Fisher) was added and incubated for 1 h at room temperature followed by PBST wash. Tetramethylbenzidine was used as a developing reagent followed by the addition of sulfuric acid to stop color development. Absorbance was measured at 450 nm. A positive control was prepared by incubating tissue lysate with 50 mM HOCl for 30 min while a negative control was a non-derivatized protein sample. For serum, samples were diluted 1:5000 with PBS–EDTA and then processed as stated above. Samples with technical replicates greater than 20% coefficient of variation were excluded in the analysis.

### Myeloperoxidase activity assay

The MPO activity assay involves the production of HOCl and its reaction with aminophenyl fluorescein (APF; Cayman Chemical, U.S.A.) to produce a fluorescent product. In brief, frozen pectoralis muscles were crushed under liquid nitrogen followed by homogenization using 0.5% hexadecyltrimethylammonium bromide in PBS. Ten microliters of MPO standards (Cayman Chemical, U.S.A.) and tissue lysates were pipetted into a 384-well plate. The enzymatic reaction was initiated by the addition of 50 μl of working solution containing 10 μM APF and 20 μM hydrogen peroxide. The change in fluorescence was measured every minute for 30 min using a fluorescent plate reader (Fluostar Optima, BMG). The excitation wavelength was 485 nm with an emission wavelength range of 515–530 nm.

### Creatine kinase activity assay

CK was measured using CK-NAC assay (Randox Laboratories, Crumlin, U.K.). Enzyme activity was measured every minute for 30 min using a spectrometer (BioTek Powerwave XS).

### Creatine and creatinine concentration in serum

Serum creatine and creatinine were measured by capillary electrophoresis based on a published method with some modifications [92]. Serum samples, creatine and creatinine standards were diluted using 50 mM phosphate buffer (pH 6.0), 0.01% v/v L-histidine with 150 mM SDS prior to analysis. The capillary was 75-μm bare fused silica with an effective length of 36.5 cm. The background electrolyte was 50 mM phosphate buffer (pH 6.0) with 150 mM SDS. Samples were injected hydrodynamically (10 mbar for 10 s), with migration induced by applying a constant +15 kV. Peak detection was performed at 200 nm. Area under the curve was analyzed using Agilent ChemStation software.

### Cibacron blue pulldown of albumin and ITIH4 in serum

Albumin and ITIH4 were enriched using Cibacron Blue 3GA beads (Sigma–Aldrich, C1535) to verify the identity of chlorinated proteins. Serum samples were diluted in PBS and incubated with 50 μl of CB beads for 1 h. After incubation, the unbound fraction was removed, and the beads were washed five times with 500 μl of PBS. Bound proteins were eluted with 1× reducing Laemmli buffer at 70°C for 10 min. Purified, unbound, and no-binding control fractions were analyzed by Western blot as described in the ‘Material and Methods’ section.

### Statistics

Statistical analysis and figure generation were performed using GraphPad Prism v10. Comparison of different age groups was performed using two-way ANOVA with Tukey’s multiple comparison test. Repeated-measures two-way ANOVA with Tukey’s multiple comparison test was used for grip tests. An unpaired *t*-test with Welch’s correction was performed when comparing the 3-month-old strains. Pearson correlation was used to determine the correlation between the biomarkers. Statistical significance was set to *P<*0.05. Cohen’s *d*, corrected for unequal sample sizes, was calculated to support differences observed between the animals; results are presented in Supplementary Figure S12.

## Supplementary Material

Supplementary Figures S1-S12

## Data Availability

All data are included in the journal article and the supplementary files. Raw data will be made available upon request.

## Open Access

Open access for this article was enabled by the participation of University of Western Australia in an all-inclusive Read & Publish agreement with Portland Press and the Biochemical Society under an agreement with CAUL.

## Abbreviations

2D-SDS/PAGE: two-dimensional sodium dodecyl sulfate polyacrylamide electrophoresis
ACN: acetonitrile
APF: aminophenyl fluorescein
BSA: bovine serum albumin
CITyr: chlorinated tyrosines
CK: creatine kinase
DMD: Duchenne muscular dystrophy
DNP: 2,4-dinitrophenyl
DNPH: 2,4-dinitrophenylhydrazine
DTT: dithiothreitol
FA: formic acid
FLM: BODIPY-FL-N-(2-aminoethyl)-maleimide
HOCl: hypochlorous acid
ITIH4: inter-alpha-trypsin inhibitor heavy chain 4
*mdx*: mouse model of dystrophy
DMD^*mdx*^: rat model of dystrophy
MPO: myeloperoxidase
MS: mass spectrometry
PBST: PBS–1% Tween 20
TCA: trichloroacetic acid
TFA: trifluoroacetic acid
TBST: Tris-buffered saline with Tween 20
