# Supplementary material for: Serum albumin oxidation and inter-alpha-trypsin-inhibitor heavy chain-4 abundance correlate with the dystropathology in the rat model of Duchenne muscular dystrophy
Source: Biochem J. 2026 Oct 27;483(11):2255–71. doi: 10.1042/BCJ20260160 (PMC13714326; doi:10.1042/BCJ20260160)
Supplement: Supplementary file 1 [file BCJ-2026-0160_supp.pdf]

Supplementary Data

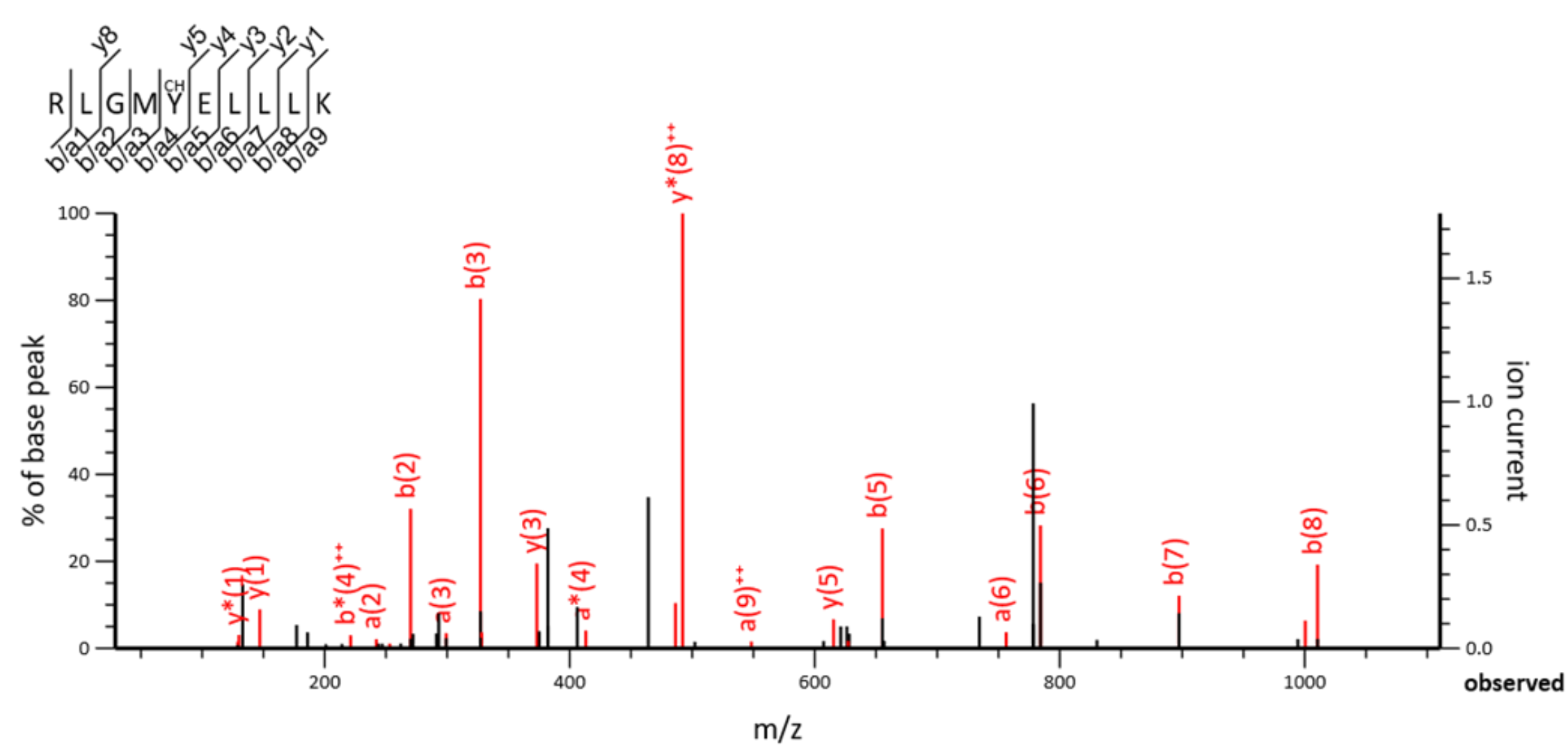

#	a	a++	a*	a*++	b	b++	b*	b*++	Seq.	y	y++	y*	y*++	#
1	129.114	65.060	112.087	56.547	157.108	79.058	140.082	70.545	R					10
2	242.198	121.602	225.171	113.089	*270.193*	135.600	253.166	127.087	L	1113.578	557.293	1096.551	548.779	9
3	299.219	150.113	282.193	141.600	*327.214*	164.111	310.187	155.597	G	1000.494	500.751	983.467	492.237	8
4	430.260	215.633	413.233	207.120	458.254	229.631	441.228	221.118	M	943.472	472.240	926.446	463.727	7
5	627.284	314.146	610.257	305.632	*655.279*	328.143	638.252	319.630	Y	812.432	406.720	795.405	398.206	6
6	756.326	378.667	739.300	370.154	*784.321*	392.664	767.295	384.151	E	615.408	308.207	598.381	299.694	5
7	869.411	435.209	852.384	426.696	*897.405*	449.206	880.379	440.693	L	486.365	243.686	469.338	235.173	4
8	982.495	491.751	965.468	483.238	*1010.490*	505.748	993.463	497.235	L	373.281	187.144	356.254	178.631	3
9	1095.579	548.293	1078.552	539.780	1123.574	562.290	1106.547	553.777	L	260.197	130.602	243.170	122.089	2
10									K	147.113	74.060	130.086	65.547	1

Supplementary 1. MS/MS spectrum and ion sequence of R.RLGMY[Cl]YELLK.V $(MH_2)^{2+}$. Cl is chlorinated modification of tyrosine. Data generated from MASCOT database. Peptide MASCOT score of 45, > 29 indicates homology, > 41 indicates identity.

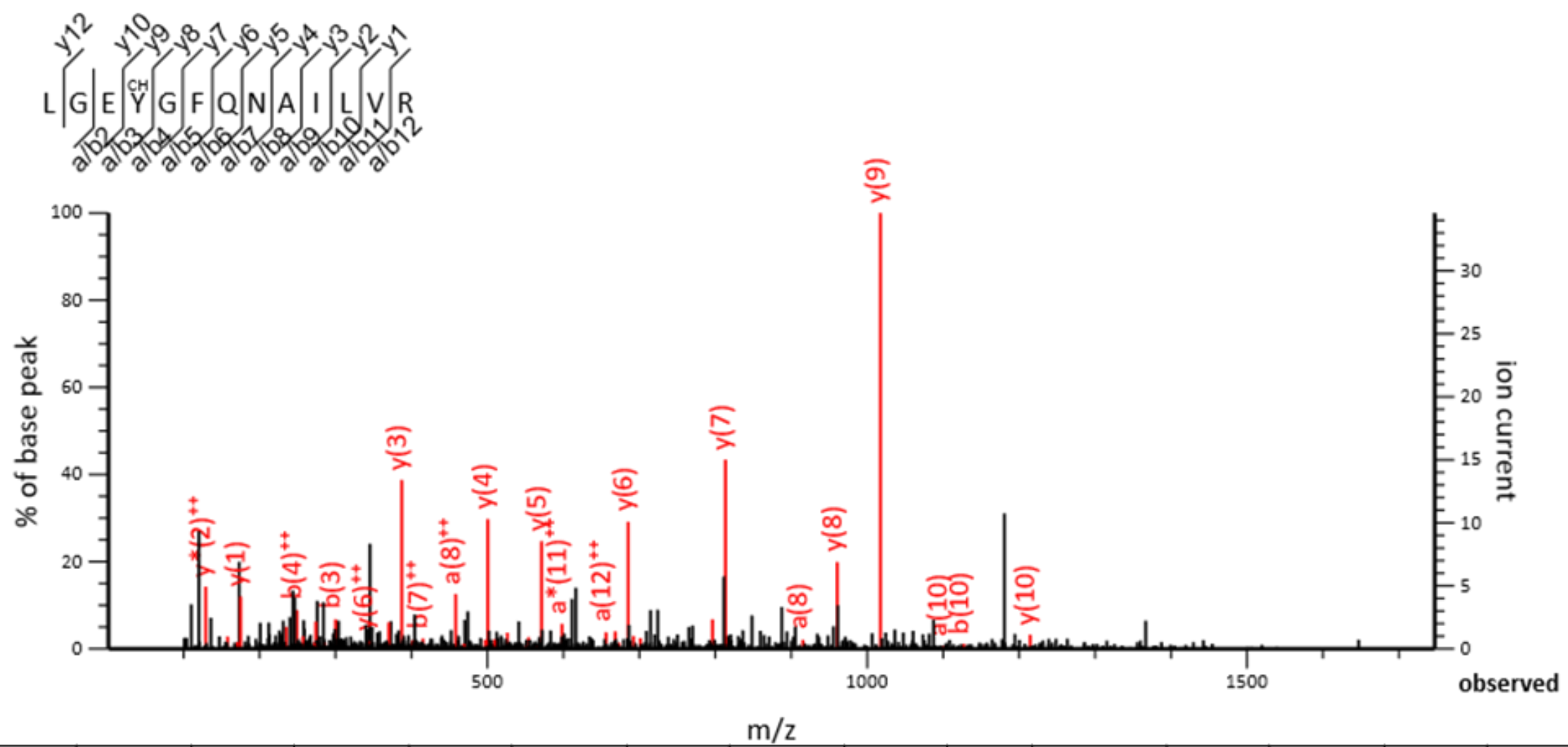

#	a	a^{++}	a*	a^{*++}	b	b^{++}	b*	b^{*++}	Seq.	y	y^{++}	y*	y^{*++}	#
1	86.0964	43.5519			114.0913	57.5493			L					13
2	143.1179	72.0626			171.1128	86.06			G	1400.6724	700.84	1383.646	692.327	12
3	272.1605	136.584			300.1554	150.581			E	1343.6509	672.329	1326.624	663.816	11
4	469.1848	235.096			497.1798	249.094			Y	1214.6083	607.808	1197.582	599.295	10
5	526.2063	263.607			554.2012	277.604			G	1017.584	509.296	1000.557	500.782	9
6	673.2747	337.141			701.2696	351.139			F	960.5625	480.785	943.536	472.272	8
7	801.3333	401.17	784.3067	392.657	829.3282	415.168	812.3017	406.655	Q	813.4941	407.251	796.4676	398.737	7
8	915.3762	458.192	898.3497	449.679	943.3711	472.189	926.3446	463.676	N	685.4355	343.221	668.409	334.708	6
9	986.4133	493.71	969.3868	485.197	1014.408	507.708	997.3817	499.195	A	571.3926	286.2	554.3661	277.687	5
10	1099.497	550.252	1082.471	541.739	1127.492	564.25	1110.466	555.737	I	500.3555	250.681	483.3289	242.168	4
11	1212.582	606.794	1195.555	598.281	1240.576	620.792	1223.55	612.279	L	387.2714	194.139	370.2449	185.626	3
12	1311.65	656.329	1294.623	647.815	1339.645	670.326	1322.618	661.813	V	274.1874	137.597	257.1608	129.084	2
13									R	175.119	88.0631	158.0924	79.5498	1

Supplementary 2. MS/MS spectrum and ion sequence of K.LGEY[Cl]GFQNAILVR.Y $(MH_2)^{2+}$. Cl is chlorinated modification of tyrosine. Data generated from MASCOT database. Peptide MASCOT score of 54, > 41 indicates homology, > 43 indicates identity.

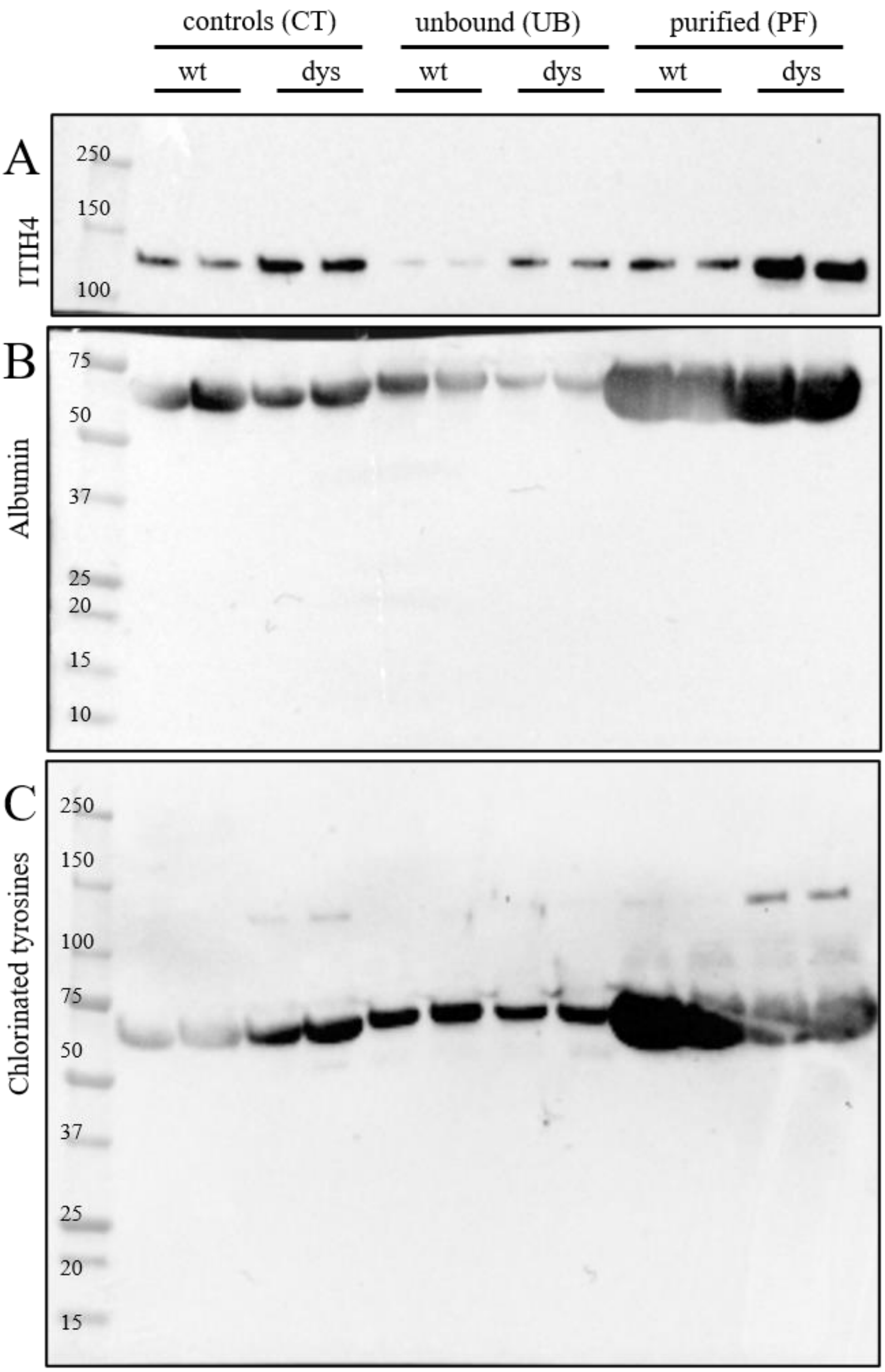

Supplementary 3. Serum albumin and inter-alpha trypsin inhibitor 4 (ITIH4) pull-down using Cibacron Blue. Albumin and ITIH4 were enriched from serum using Cibacron Blue beads, yielding a purified fraction (PF) with markedly higher levels of both proteins compared with the unbound (UB) fractions and control samples (CT). Western blot membranes of (A) ITIH4, (B) albumin, and (C) chlorinated tyrosines. Rat serum samples were obtained from 3-month-old wild-type (WT) and dystrophic (Dys) rats (n = 2 per group).

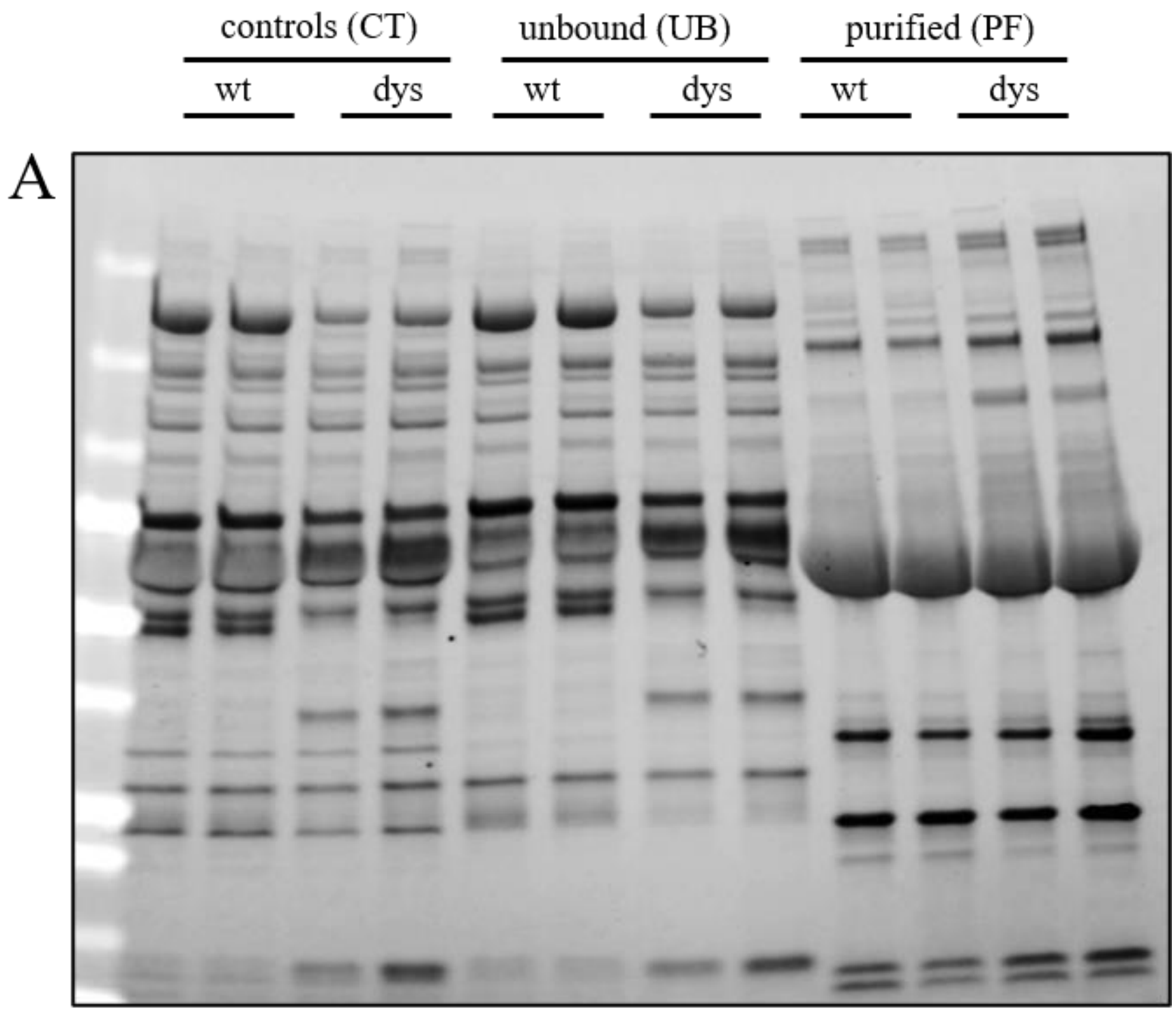

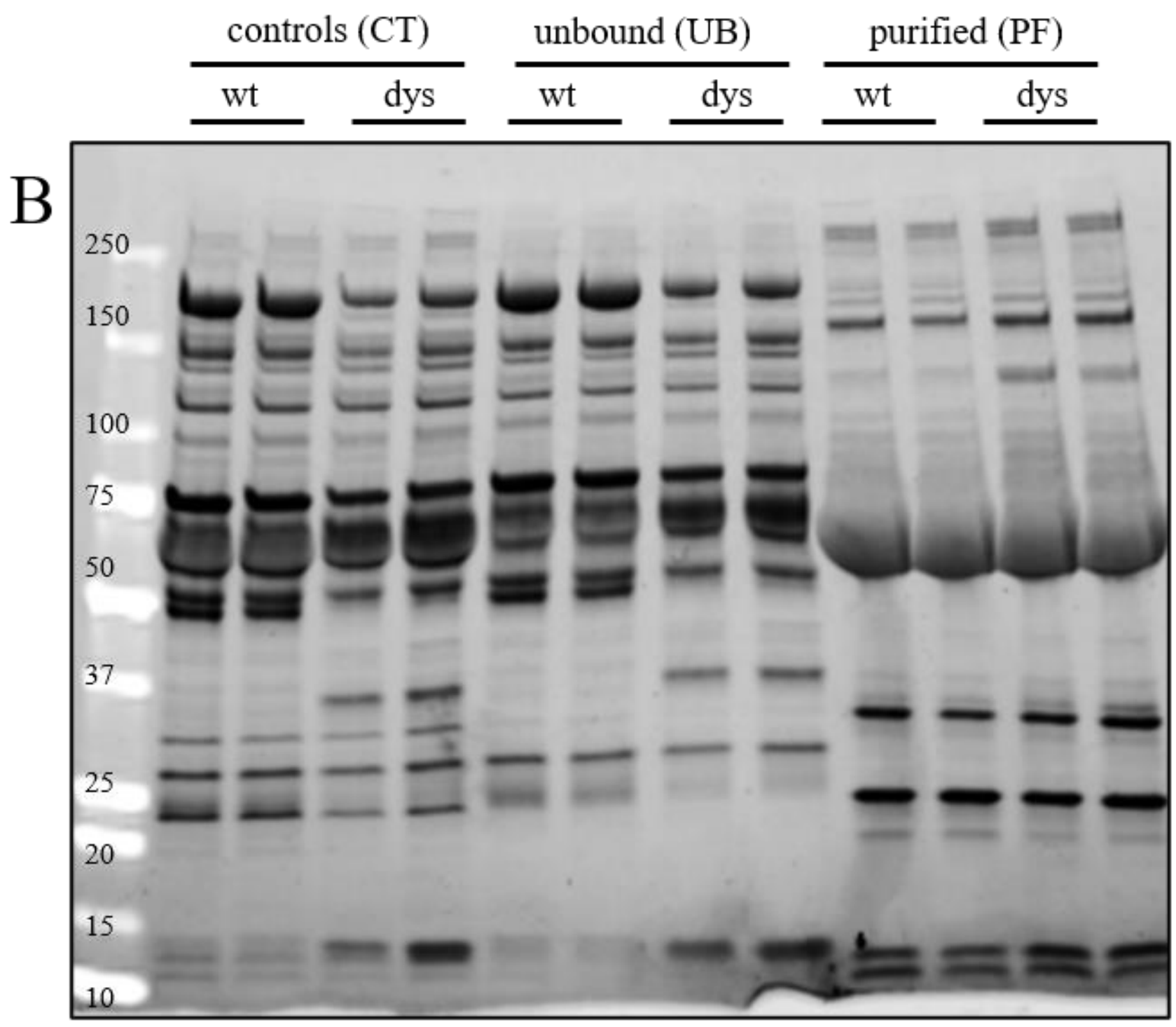

Supplementary 4. Serum albumin and inter-alpha trypsin inhibitor 4 pulldown using Cibacron blue. Total protein signal corresponding to membranes for western blot detection in Supp. 3 of (A) ITIH4 and Albumin, (B) chlorinated tyrosines. Rat serum from 3-month-old wildtype (wt) and dystrophic rats (dys), n=2.

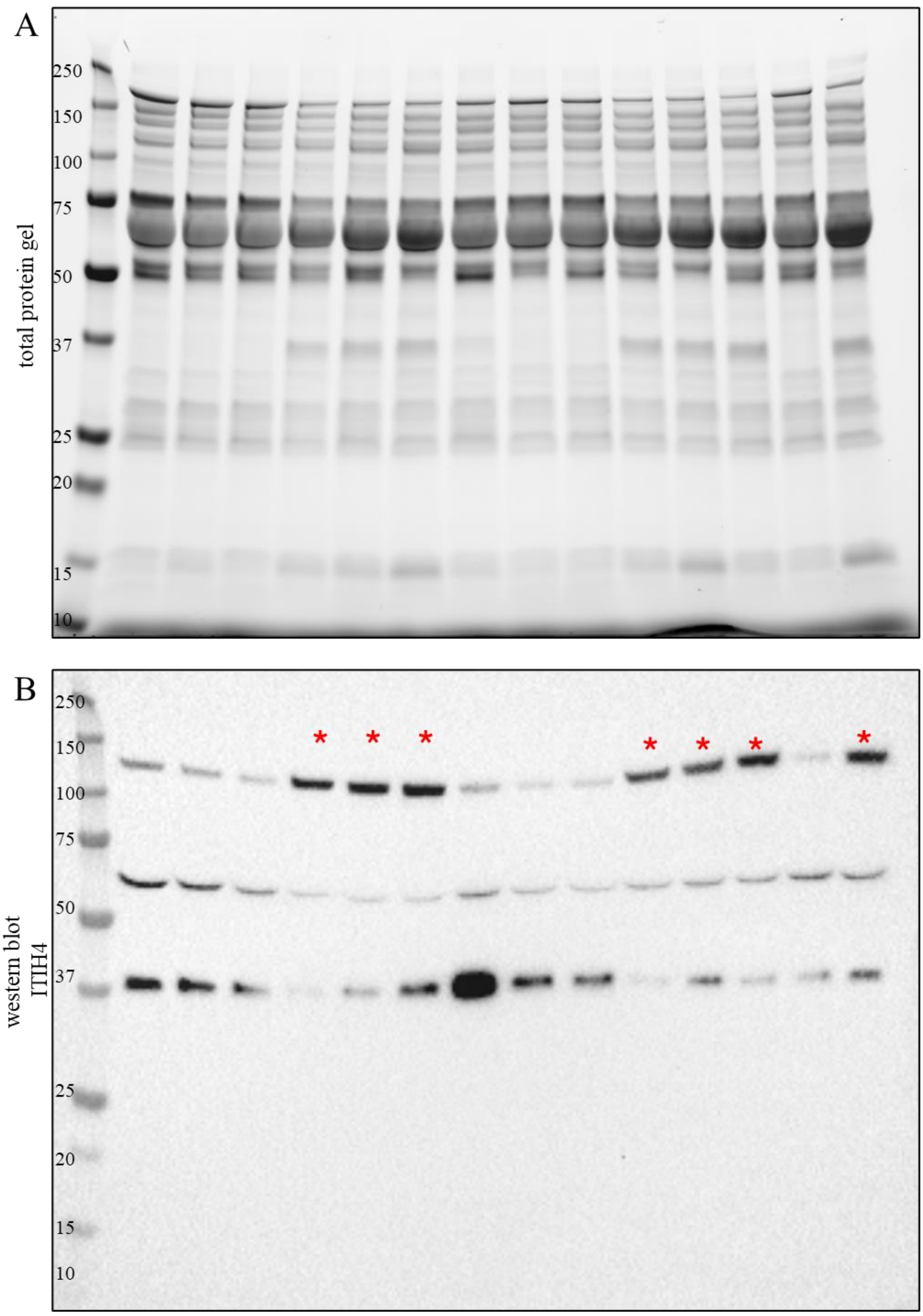

Supplementary 5. Serum intact ITIH4 (120 kDa) abundance in 3-months old rats. (A) total protein gel, (B) Inter-alpha trypsin inhibitor 4 (ITIH4) western blot. (*) represent dystrophic samples.

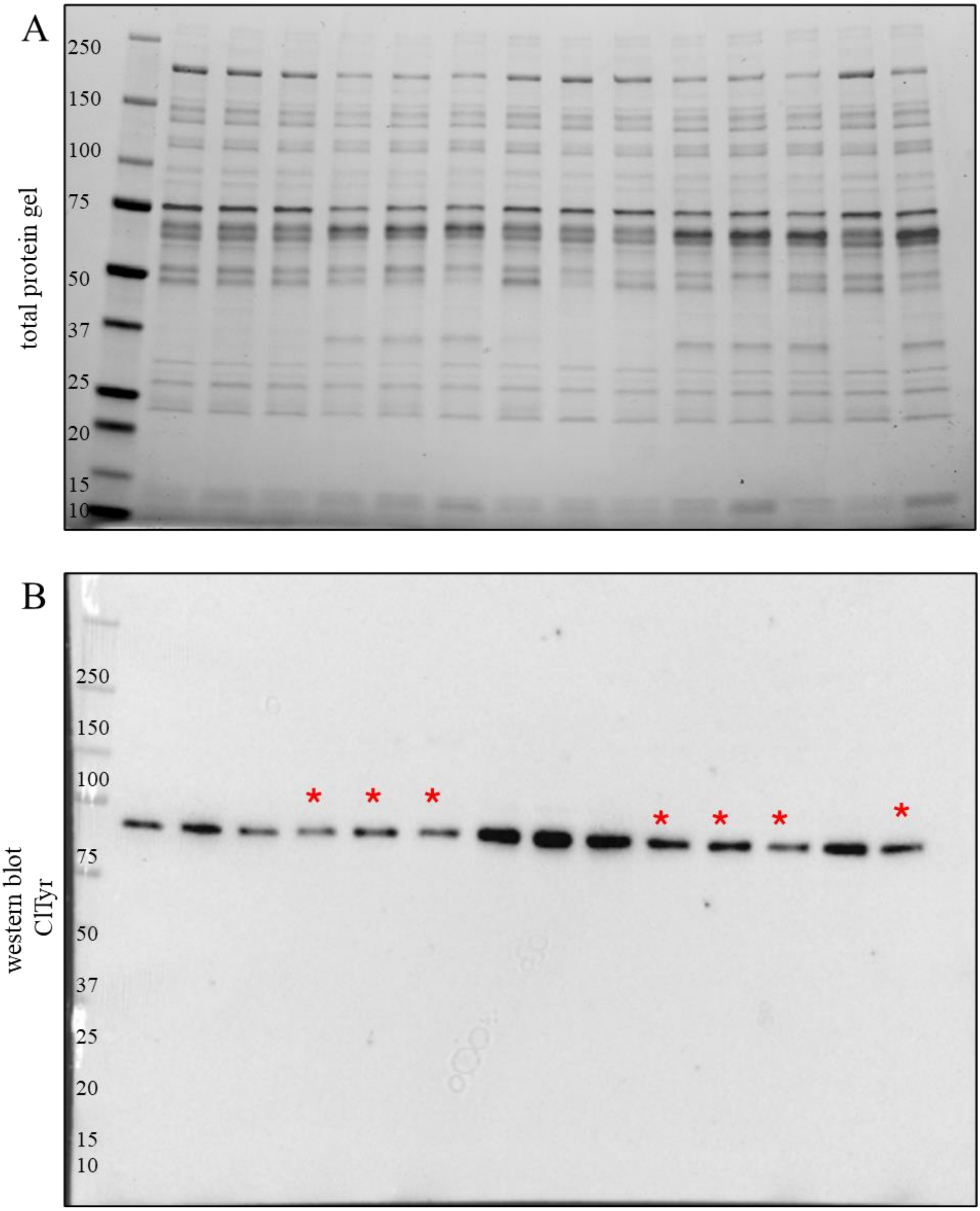

Supplementary 6. Serum albumin chlorination in 3-months old rats. (A) total protein gel, (B) chlorotyrosine western blot. (*) represent dystrophic samples.

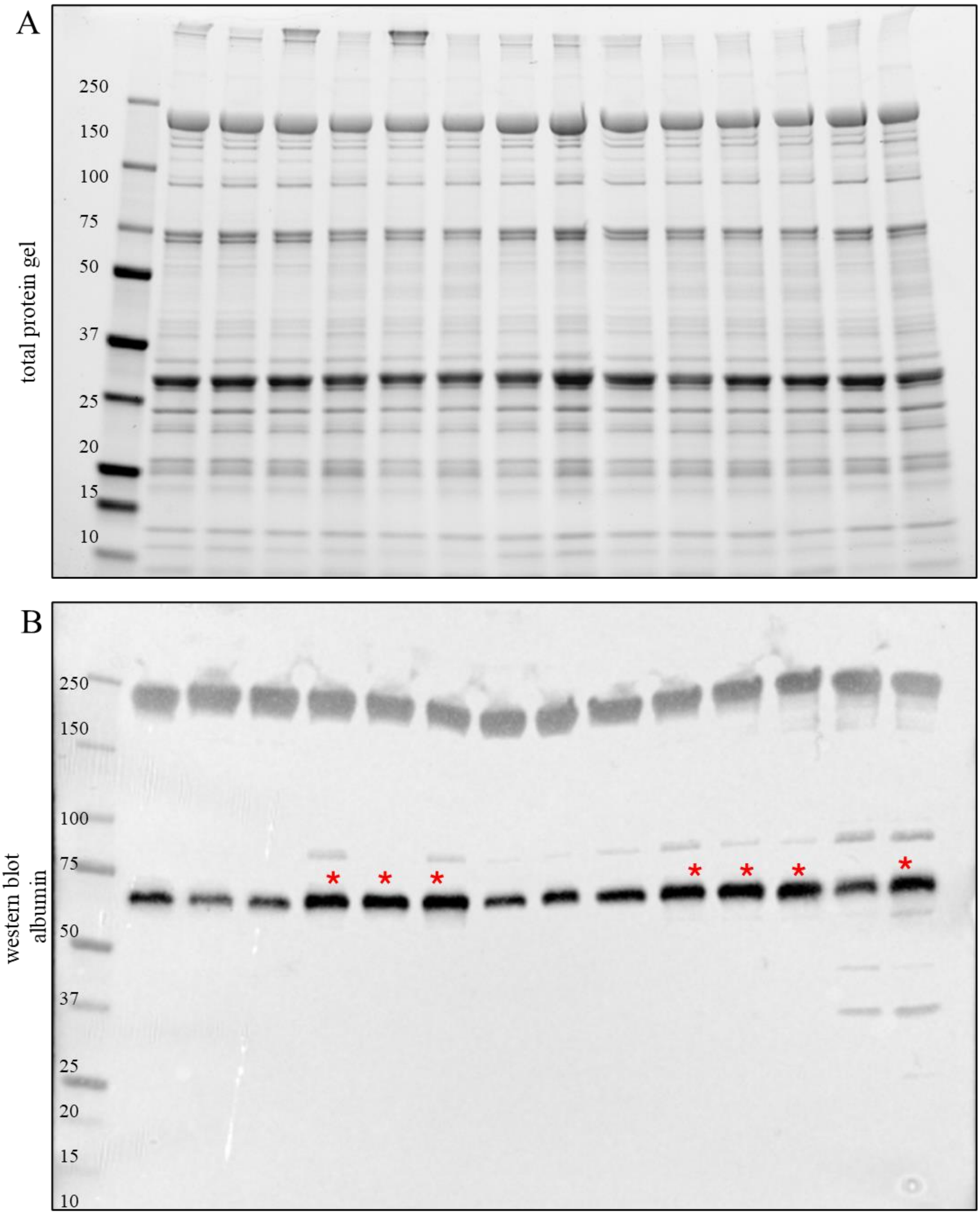

Supplementary 7. Muscle albumin abundance in 3-months old rats. (A) total protein gel, (B) albumin western blot. (*) represent dystrophic samples.

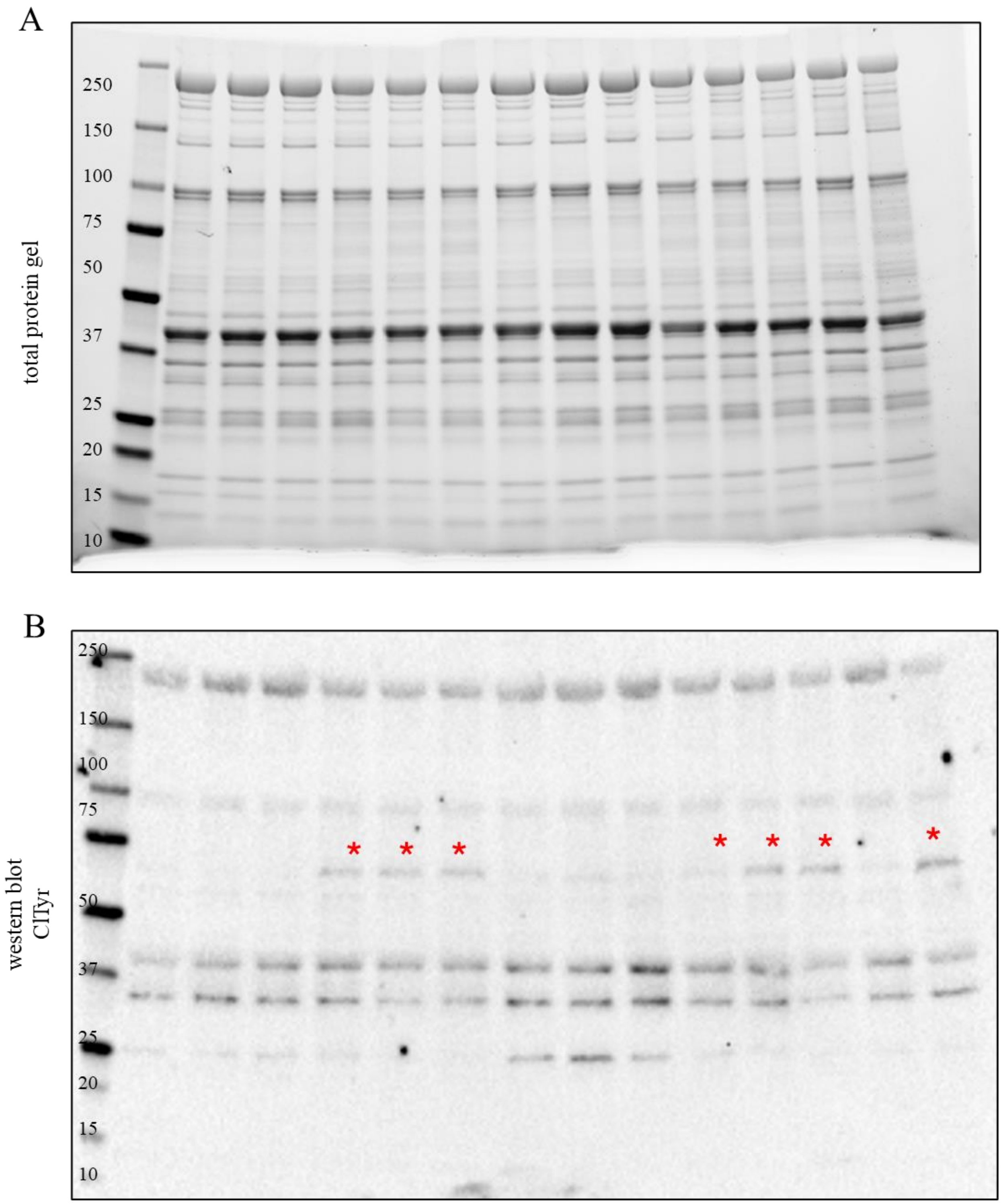

Supplementary 8. Muscle chlorinated protein tyrosines in 3-months old rats. (A) total protein gel, (B) chlorotyrosine western blot. (*) represent dystrophic samples.

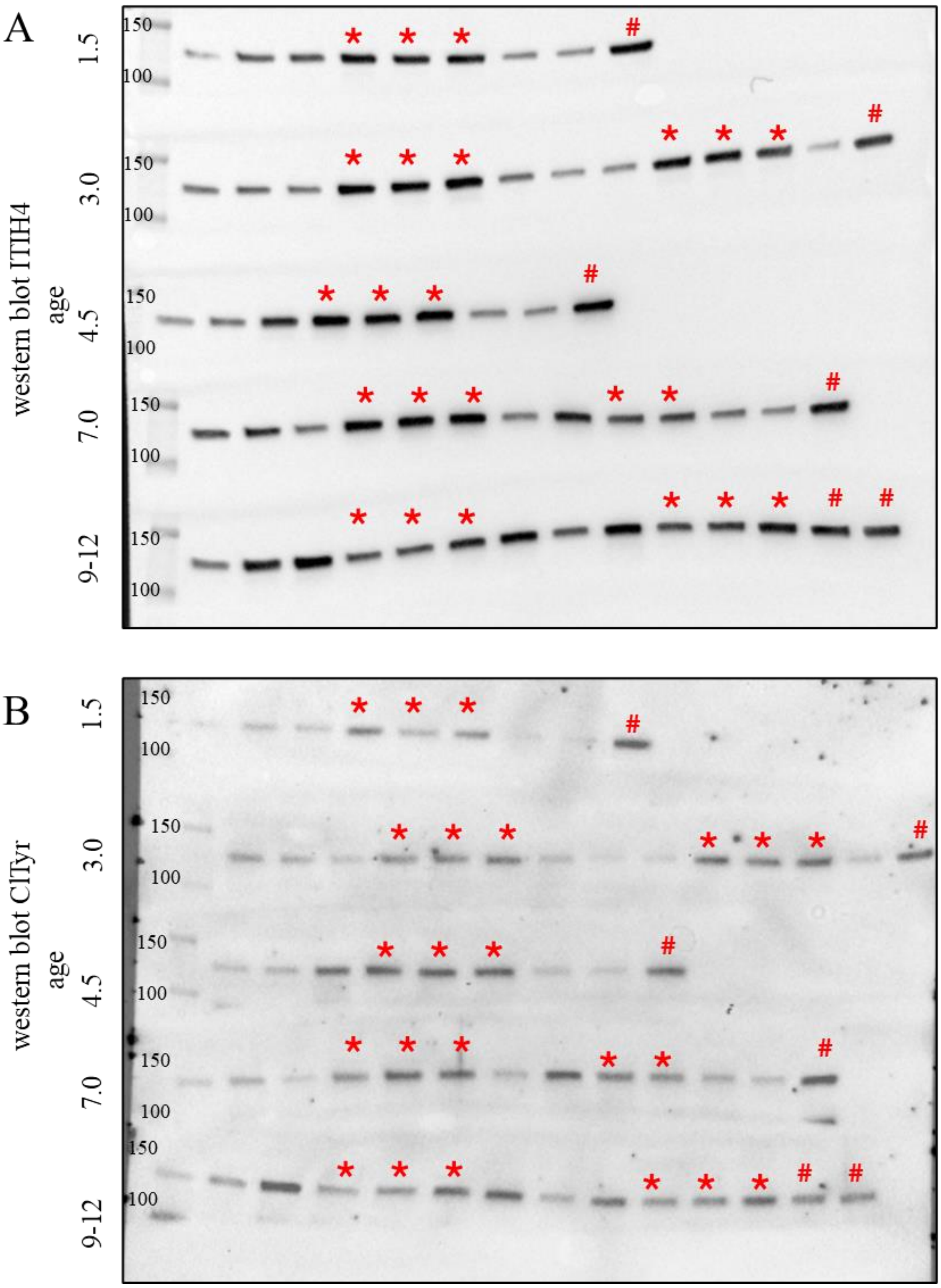

Supplementary 9. Rat serum (A) ITIH4 and (B) chlorotyrosine (ClTyr) western blot ages 1.5 – 12 months. (*) represent dystrophic samples, (#) is a common sample loaded across the different gels.

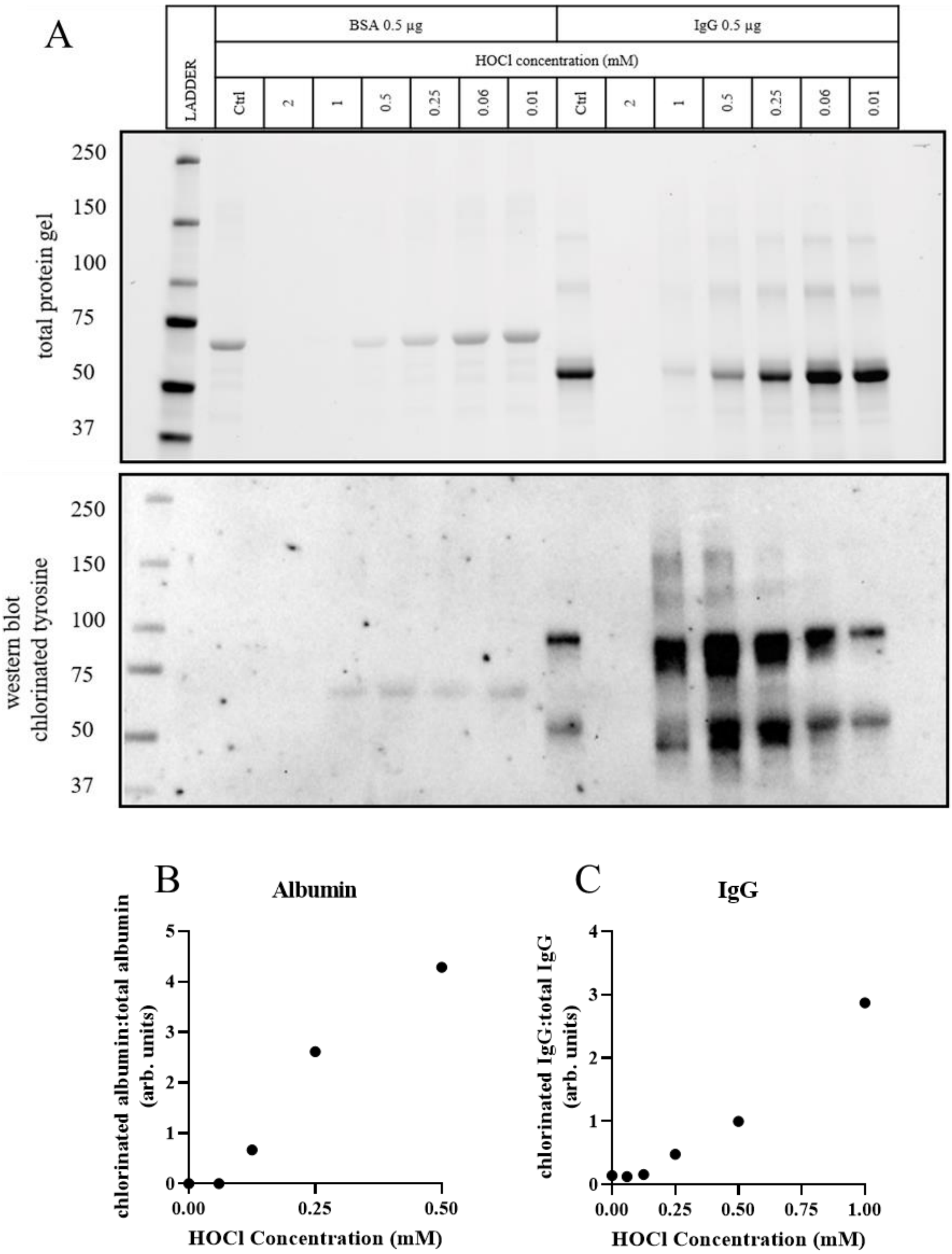

Supplementary 10. Validation of Hycult chlorotyrosine antibody (HP5002). Human serum albumin and immunoglobulin (IgG) protein standards were incubated with increasing concentrations of hypochlorous acid (HOCl). (A) total protein gel and companion chlorotyrosine western blot. (B) ratio of chlorinated albumin to total albumin, (C) ratio of chlorinated IgG to total IgG. For both B and C, increase in chlorination observed with increasing HOCl concentration. Note: decrease in intensity of the protein standards in the total protein gel may indicate protein degradation induced by HOCl.

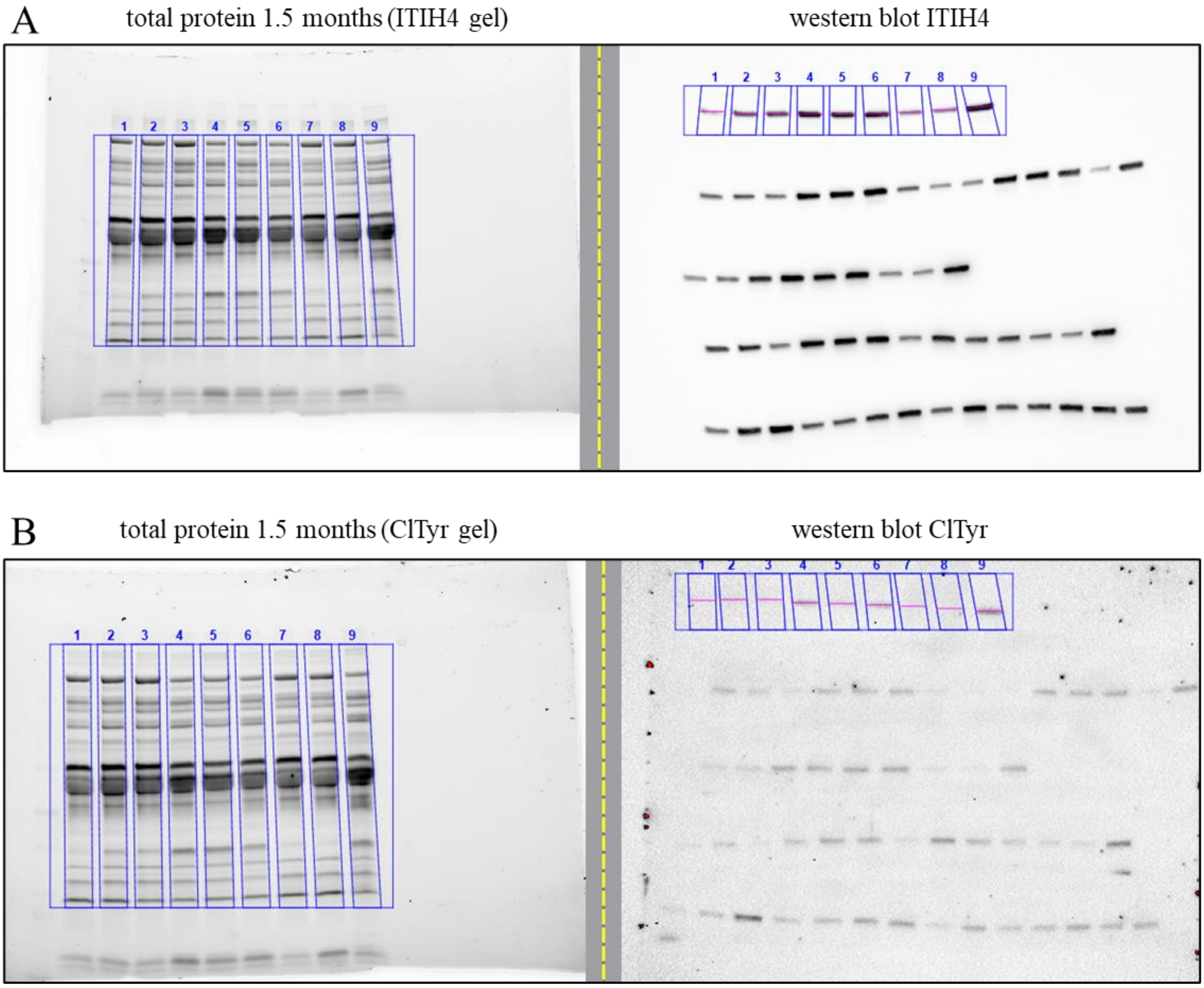

Supplementary 11. Ratiometric analysis of chlorinated ITIH4. (A) ITIH4 western blot signal was normalised to total protein signal, (B) ClTyr western blot was normalised to total protein signal. Ratio of normalised ClTyr and ITIH4 (ClTyr:ITIH4) was then calculated, these values were presented in *Results f, Figure 8C*.

Serum albumin thiol oxidation

	wt				dys						
Age	mean	sd	n		mean	sd	n		pooled SD	Cohen's d	effect size
1.5	41.51	2.14	5		49.25	3.90	3		2.85	2.72	Very large
3	39.09	3.20	8		49.67	6.72	7		5.13	2.06	Very large
4.5	46.14	5.15	3		53.86	5.32	3		5.23	1.48	Very large

Serum ITIH4 abundance

	wt				dys						
Age	mean	sd	n		mean	sd	n		pooled SD	Cohen's d	effect size
1.5	0.40	0.10	5		0.83	0.16	3		0.12	3.50	Very large
3	0.38	0.08	7		0.97	0.19	7		0.15	4.00	Very large
4.5	0.48	0.10	5		1.13	0.06	3		0.09	7.37	Very large
7	0.65	0.25	7		1.04	0.20	5		0.23	1.71	Very large
9-12	0.91	0.26	6		1.01	0.13	6		0.20	0.47	Small

Serum chlorinated ITIH4

	wt				dys						
Age	mean	sd	n		mean	sd	n		pooled SD	Cohen's d	effect size
1.5	0.34	0.31	5		0.82	0.21	3		0.28	1.72	Very large
3	0.60	0.43	7		0.84	0.28	7		0.36	0.67	Medium
4.5	0.46	0.12	5		0.82	0.13	3		0.12	2.93	Very large
7	0.69	0.40	7		0.94	0.32	5		0.37	0.66	Medium
9-12	0.79	0.45	6		0.82	0.16	6		0.34	0.09	no effect

Serum total carbonylation

	wt				dys						
Age	mean	sd	n		mean	sd	n		pooled SD	Cohen's d	effect size
1.5	1.23	0.23	5		1.02	0.29	3		0.25	0.83	Large
3	0.81	0.26	8		0.58	0.26	8		0.26	0.90	Large
4.5	1.03	0.12	5		0.47	0.44	3		0.27	2.07	Very large

Tissue protein thiol oxidation

	wt				dys						
Age	mean	sd	n		mean	sd	n		pooled SD	Cohen's d	effect size
1.5	19.19	3.05	8		26.86	5.42	8		4.40	1.74	Very large
3	19.90	3.89	8		26.96	3.37	8		3.64	1.94	Very large
4.5	16.99	2.75	7		25.24	7.37	7		5.56	1.48	Very large
7	17.68	2.75	8		24.78	4.15	8		3.52	2.02	Very large
9-12	19.13	2.71	7		25.22	5.38	8		4.36	1.40	Very large

Tissue protein carbonylation

	wt				dys						
Age	mean	sd	n		mean	sd	n		pooled SD	Cohen's d	effect size
1.5	1.09	0.18	8		1.34	0.16	7		0.17	1.45	Very large
3	0.98	0.15	7		1.24	0.18	7		0.16	1.60	Very large
4.5	1.15	0.40	7		1.20	0.34	7		0.37	0.15	no effect
7	0.96	0.16	8		1.05	0.23	8		0.20	0.46	Small
9-12	0.85	0.15	5		1.09	0.24	7		0.21	1.12	Very large

Tissue protein tyrosine halogenation

	wt				dys						
Age	mean	sd	n		mean	sd	n		pooled SD	Cohen's d	effect size
1.5	1.50	0.36	8		1.89	0.34	8		0.35	1.13	Very large
3	1.49	0.23	7		1.82	0.29	8		0.26	1.24	Very large
4.5	1.66	0.27	8		1.43	0.15	6		0.23	1.01	Very large
7	1.29	0.18	8		1.45	0.32	8		0.26	0.62	Medium
9-12	1.22	0.24	8		1.20	0.29	8		0.27	0.06	no effect

Supplementary 12. Cohen's D calculation for serum and tissue oxidised proteins. Cohen's D effect size classification: no effect (<0.2), small (0.2-0.49), medium (0.5-0.79), large (0.8–0.99), very large (≥1.0).
